# Phylogeography of sharks and rays: a global review based on life history traits and biogeographic partitions

**DOI:** 10.7717/peerj.15396

**Published:** 2023-06-01

**Authors:** Sudha Kottillil, Chetan Rao, Brian W. Bowen, Kartik Shanker

**Affiliations:** 1 Centre for Ecological Sciences, Indian Institute of Science, Bengaluru, Karnataka, India; 2 Department of Energy and Environment, TERI School of Advanced Studies, New Delhi, India; 3 Dakshin Foundation, Bengaluru, Karnataka, India; 4 Hawai‘i Institute of Marine Biology, University of Hawaii, Kaneohe, Hawai‘i, United States of America

**Keywords:** Conservation genetics, Elasmobranchs, Genetic diversity, Mitochondrial DNA, Population structure, Sharks, Rays, Phylogeography, Life history

## Abstract

Considerable research exists on the life history traits, evolutionary history, and environmental factors that shape the population genetic structure of marine organisms, including sharks and rays. Conservation concerns are particularly strong for this group as they are highly susceptible to anthropogenic stressors due to a combination of life history traits including late maturity and low fecundity. Here, we provide a review and synthesis of the global phylogeography of sharks and rays. We examined existing data for 40 species of sharks belonging to 17 genera and 19 species of rays belonging to 11 genera. Median joining haplotype networks were constructed for each species for the mtDNA cytochrome C oxidase subunit I (COI), and an Analysis of Molecular Variance (AMOVA) was conducted to understand patterns of genetic diversity and structure across the three major ocean basins—the Indian, Atlantic and Pacific Oceans. Haplotype networks showed very shallow coalescence in most species, a finding previously reported for marine teleosts. Star topologies were predominant among sharks while complex mutational topologies predominated among rays, a finding we attribute to extremely limited dispersal in the early life history of rays. Population structuring varied amongst species groups, apparently due to differences in life history traits including reproductive philopatry, site fidelity, pelagic habitat, migratory habits, and dispersal ability. In comparison to reef-associated and demersal species, pelagic and semi pelagic species showed lower levels of structure between and within ocean basins. As expected, there is variation between taxa and groups, but there are also some broad patterns that can guide management and conservation strategies.

## Introduction

Many marine organisms are characterized by very large distribution ranges, a finding often attributed to the lack of physical barriers. Allen (2008) estimated that the average range of a teleost (bony) reef fish in the Indo–Pacific is 9 million km^2^, roughly the size of China, compared to 350,000 km^2^ for a typical freshwater fish range (Albert & Carvalho, 2011). The spatial scales of population structure and dispersal in marine ecosystems are also much larger than in terrestrial and freshwater environments (Avise, 2000). Long-distance colonisation and range expansion, both facilitated and constrained by oceanographic and geographic processes, have shaped the distribution and genetic architecture of marine fishes (Hellberg, 2009).

The patterns of genetic variation within species are linked to geographical processes that give rise to sub-divided populations, as indicated by quantifiable factors such as genetic connectivity and demography. The study of population connectivity is especially pertinent to management practices for commercial exploitation and conservation (Allendorf, Luikart & Aitken, 2013). Genetic diversity is also an important axis for species assessment in a conservation context, especially for wide-ranging marine species like sharks (Domingues, Hilsdorf & Gadig, 2018).

Sharks and rays play a crucial role in the sea by maintaining coastal and oceanic ecosystem structure and function. Large sharks function as top predators while smaller sharks are mesopredators and prey of larger sharks and other oceanic predators (Dulvy et al., 2008). Unlike teleosts, most elasmobranchs (sharks, rays, and skates) show late sexual maturity, long gestation period, low fecundity, slow growth rate, high level of maternal investment and long-life spans (Dulvy et al., 2014). These extreme life histories result in elasmobranchs being among the slowest reproducing vertebrates in the ocean and make their populations extremely vulnerable to anthropogenic pressures such as overfishing, habitat modification, pollution and climate change (Heist, 2008; Snelson, Burgess & Roman, 2008). The primary cause of declining shark and ray populations is overfishing, as harvest rates exceed their capacity to replenish, and their life history traits render them vulnerable to rapid declines (Bräutigam et al., 2015; Dulvy et al., 2021; Pacoureau et al., 2021). More than half of this fishing mortality is due to bycatch (Hall, Alverson & Metuzals, 2000; Gupta et al., 2020; Dulvy et al., 2021). The expansion of the shark and ray fishing industry is an outcome of declining commercial fish populations (teleosts) and/or stringent restrictions on their capture (Bräutigam et al., 2015).

Sharks are found in coastal, demersal, and pelagic habitats that are largely limited to continental shelves, although there are a few completely oceanic species like *Carcharhinus longimanus* (oceanic whitetip shark). Several species in the family Sphyrnidae (hammerheads), *Carcharhinus falciformis* (silky shark), *Galeocerdo cuvier* (tiger shark), and *Carcharodon carcharias* (white shark) migrate between coastal and oceanic waters (Ferretti et al., 2010). Rays are mostly marine except for a few species in the family Dasyatidae capable of living in low salinity habitats, and members of Potamotrygonidae completely adapted to a life cycle in freshwater (Last et al., 2016). Like sharks, they occupy a variety of niches with pelagic rays capable of undertaking long migrations (Last et al., 2016). However, population subdivisions may be more common in rays because of limited dispersal and greater susceptibility to geographical impediments (Heist, 2005; Heupel, Carlson & Simpfendorfer, 2007). Sharks that inhabit coastal waters aggregate for mating and parturition at specific discrete locations which provide protection for juveniles (Heupel, Carlson & Simpfendorfer, 2007). The extent of population subdivision and genetic divergence between populations in different geographic regions is directly influenced by such segregation and philopatry (Hueter et al., 2005) and the dispersal ability of individuals.

For example, philopatry to natal sites in blacktip reef sharks (*Carcharhinus melanopterus*) is a major contributor to genetic structuring within the Indo-Pacific and between islands in French Polynesia, by reducing dispersal (Mourier & Planes, 2013; Vignaud et al., 2014). Bull shark juveniles from nurseries in the Gulf of Mexico and Atlantic showed significant genetic variation in the mitochondrial control region (mtDNA-CR) but were homogenous with nuclear microsatellites indicating male biased dispersal (Laurrabaquio et al., 2019). Similarly, population structure (using mtNADH sequences) among juvenile bull sharks from 13 nurseries located in rivers around Northern Australia also indicated a strong influence of female reproductive philopatry (Tillett et al., 2012). These observed genetic differences support philopatry and indicate a strong role in shaping population separations in bull sharks (Tillett et al., 2012; Laurrabaquio et al., 2019). Site fidelity and long-term residency also resulted in fine-scale genetic structuring within reef manta rays (*Mobula alfredi*) in New Caledonia (Lassauce et al., 2022).

Understanding elasmobranch biology and life history is therefore important for evolving species-specific management plans. Their governance poses a challenge as many species of sharks and rays migrate across national boundaries and international waters, and there is little knowledge/information about the migratory habits of transboundary species in international waters (Bräutigam et al., 2015; Kizhakudan et al., 2015). Presently, conservation measures for sharks and rays are influenced by political boundaries, oceanic expanses, and centres of high demand (Bräutigam et al., 2015). Conservation efforts are under-resourced due to lack of adequate funds, technical capacity and political will to efficiently monitor, control and manage elasmobranch fisheries/trade (Bräutigam et al., 2015).

Hence, we examined patterns of phylogeography and population structure within and across multiple families of sharks (Carcharhinidae, Cetorhinidae, Hemiscyllium, Odontaspididae, Stegostomatidae, Alopiidae, Rhincodontidae, Sphyrnidae and Lamnidae) and rays (Dasyatidae, Mobulidae, Myliobatidae and Gymnuridae) in relation to their habitat and life history. We explored these patterns by (a) compiling data from a variety of published and unpublished sources, (b) constructing haplotype networks and examining network topology, (c) estimating nucleotide and haplotype diversity, and (d) assessing population genetic structure using AMOVA. In a few species, we report data from a single study, but for many species, our meta-analysis combines data from multiple sources, both published and unpublished, and provides insights from comparisons across genera, and between sharks and rays.

## Methods

A literature review was carried out on the phylogeography of shark and ray species to check for availability of sequence data. The accession numbers provided in publications were used to identify sequences from GenBank (Benson et al., 2013), a National Centre for Biological Sciences (National Center for Biotechnology Information, 1988) and US National Institute of Health (NIH) sequence database, as well as the geographical locations of specimens. Additional unpublished sequences were downloaded from GenBank and included in the study. We then narrowed the species list to those with sufficient sample sizes (>10). Cytochrome C oxidase subunit I (COI) was selected for this analysis because it was the most common sequence across species. Other markers may provide greater resolution for the detection of intraspecific (population level) divergences but it is a pragmatic choice based on data availability. Our final set of target species included 40 shark species from 17 genera (Table 1) and 19 ray species from 11 genera (Table 2). All mitochondrial DNA sequences (mtDNA) were obtained/downloaded from GenBank (Tables S1 and S2). The sample collection locations provided in the published research article and/or on GenBank were used for population analysis.

**Table 1:** Life history attributes and distribution of shark species assessed in the present study.

**Species name**	**Common name**	**Distribution**	**Habitat**	**Body size** ^a^ **(cm)**	**IUCN category**	**Traits** ^b^
*Alopias pelagicus*	Pelagic thresher shark	Indo–Pacific	Oceanic and pelagic	259–376	Endangered	No evidence of philopatry; ovoviviparous
*Alopias superciliosus*	Bigeye thresher shark	Global	Coastal, oceanic and pelagic	470–484	Vulnerable	No evidence of philopatry; ovoviviparous
*Alopias vulpinus*	Common thresher shark	Global	Coastal, oceanic and pelagic	575–635	Vulnerable	No evidence of philopatry; ovoviviparous
*Carcharhinus altimus*	Bignose shark	Global	Benthopelagic	300	Near threatened	Viviparous
*Carcharhinus amblyrhynchoides*	Graceful shark	Indo–west Pacific	Coastal and pelagic	178–190	Near threatened	Viviparous
*Carcharhinus amboinensis*	Pigeye shark	Global	Coastal, oceanic and demersal	280	Data deficient	Residency, regional philopatry; viviparous
*Carcharhinus brevipinna*	Spinner shark	Global	Coastal and pelagic	280–283	Near threatened	Regional philopatry, site fidelity; viviparous
*Carcharhinus dussumieri*	Whitecheek shark	Indo–west Pacific	Coastal and demersal	100–110	Endangered	Viviparous
*Carcharhinus falciformis*	Silky shark	Global	Coastal, oceanic and pelagic	330–350	Vulnerable	Site fidelity; viviparous
*Carcharhinus leucas*	Bull shark	Global	Coastal & estuarine and reef-associated	340–360	Near threatened	Residency, regional philopatry and site fidelity; viviparous
*Carcharhinus limbatus*	Common blacktip shark	Global	Coastal and reef-associated	258–297	Near threatened	Seasonal residency, regional philopatry and site fidelity; viviparous
*Carcharhinus longimanus*	Oceanic whitetip shark	Global	Oceanic and pelagic	350–395	Critically endangered	Seasonal residency and site fidelity; viviparous
*Carcharhinus macloti*	Hardnose shark	Indo–west Pacific	Reef-associated and demersal	100–110	Near threatened	Viviparous
*Carcharhinus melanopterus*	Blacktip reef shark	Indo–Pacific	Coastal and reef-associated	160 ≤ 200	Vulnerable	Residency, site fidelity, natal philopatry; viviparous
*Carcharhinus plumbeus*	Sandbar shark	Global	Coastal and benthopelagic	200–300	Near threatened	Seasonal residency, site fidelity; viviparous
*Carcharhinus sealei*	Blackspotted shark	Indo–west Pacific	Reef-associated	100	Near threatened	Viviparous
*Carcharhinus sorrah*	Spot-tail shark	Indo–west Pacific	Coastal and reef-associated	160–180	Near threatened	Residency; viviparous
*Carcharodon carcharias*	Great white shark	Global	Oceanic and pelagic	400–600	Vulnerable	Ovoviviparous (oophagus)
*Carcharias taurus*	Sand tiger	Atlantic, Indo–west Pacific	Coastal, reef-associated and demersal	320	Vulnerable	Seasonal residency, site fidelity; ovoviviparous
*Cetorhinus maximus*	Basking shark	Atlantic, Pacific and Artic	Coastal, oceanic and pelagic	1,200–1,220	Endangered	Ovoviviparous (oviphagy)
*Chiloscyllium griseum*	Grey bamboo shark	Indo–west Pacific	Reef-associated and demersal	77	Vulnerable	Oviparous
*Chiloscyllium indicum*	Slender bamboo shark	Indo–west Pacific	Coastal and demersal	65	Vulnerable	Oviparous
*Chiloscyllium punctatum*	Brown banded bamboo shark	Indo–west Pacific	Reef-associated and demersal	104	Near threatened	Oviparous
*Galeocerdo cuvier*	Tiger shark	Global	Coastal, oceanic and semi pelagic	400 ≥ 550	Near threatened	Residency, site fidelity; ovoviviparous
*Isurus oxyrinchus*	Short mako shark	Global	Coastal, oceanic and pelagic	200–400	Endangered	Site fidelity; ovoviviparous
*Isurus paucus*	Longfin mako	Global	Oceanic, pelagic	417–430	Endangered	Site fidelity; ovoviviparous (oviphagy)
*Negaprion acutidens*	Sicklefin lemon shark	Indo–Pacific	Coastal and demersal	310–380	Vulnerable	Residency, site fidelity; viviparous
*Negaprion brevirostris*	Lemon shark	Atlantic, East Pacific	Coastal, reef-associated and demersal	250–300	Near threatened	Residency, site fidelity, natal philopatry; viviparous
*Prionace glauca*	Blue shark	Global	Oceanic and pelagic	383–385	Near threatened	Viviparous
*Rhincodon typus*	Whale shark	Global	Coastal, oceanic and semi pelagic	1,600–2,100	Endangered	Site fidelity; ovoviviparous
*Rhizoprionodon oligolinx*	Grey sharpnose shark	Indo–west Pacific	Coastal, reef-associated	70	Least concern	Viviparous
*Rhizoprionodon acutus*	Milk shark	Indo–west Pacific	Benthopelagic	178–180	Vulnerable	Viviparous
*Scoliodon laticaudus*	Spadenose shark	Indo–west Pacific	Coastal and demersal	74–75	Vulnerable	Viviparous
*Sphyrna lewini*	Scalloped hammerhead shark	Global	Semi oceanic and semi pelagic	370–420	Critically endangered	Seasonal residency, regional philopatry and site fidelity; viviparous
*Sphyrna mokarran*	Great hammerhead shark	Global	Semi oceanic and semi pelagic	550–610	Critically endangered	Site affinity; viviparous
*Sphyrna zygaena*	Smooth hammerhead shark	Global	Semi oceanic and semi pelagic	370–400	Vulnerable	Regional philopatry; viviparous
*Stegostoma fasciatum*	Zebra shark	Not mapped	Coastal and demersal	235–246	Endangered	Seasonal residency and site fidelity; oviparous
*Triaenodon obesus*	Whitetip reef shark	Indo–Pacific	Coastal and reef-associated	170–213	Vulnerable	Residency; viviparous
*Lamna ditropis*	Salmon shark	North Pacific	Coastal, oceanic and pelagic	221–305	Least concern	Residency, seasonal residency, site fidelity, regional philopatry; ovoviviparous (oophagus)
*Lamna nasus*	Porbeagle	North Atlantic and Southern hemisphere	Oceanic, coastal and pelagic	350–365	Vulnerable	Ovoviviparous (oophagus)

**Notes.**

a Body size refers to the maximum total length of the adult shark. Total length is the length measured from the tip of the snout to the tip of the longer lobe of the caudal fin.

b Traits refer to the type of philopatry and reproduction shown by the particular shark species.

**Table 2:** Life history attributes and distribution of ray species assessed in the present study.

**Species name**	**Common name**	**Distribution**	**Habitat**	**Disc width** ^a^ **(cm)**	**IUCN category**	**Traits** ^b^
*Aetobatus narinari*	Spotted eagle ray	Atlantic Ocean	Oceanic, coastal and benthopelagic	330	Near threatened	Site fidelity; ovoviviparous
*Aetobatus ocellatus*	Ocellated eagle ray	Indo–west Pacific	Oceanic, coastal and benthopelagic	300	Vulnerable	Ovoviviparous
*Brevitrygon imbricata*	Bengal whipray	Northern Indian Ocean	Coastal and demersal	23	Data deficient	Ovoviviparous
*Brevitrygon walga*	Scaly whipray/ Dwarf whipray	Western Indian Ocean	Coastal and demersal	32	Near threatened	Ovoviviparous
*Gymnura micrura*	Smooth butterfly ray	Atlantic Ocean	Coastal and demersal	110	Data deficient	Ovoviviparous
*Gymnura poecilura*	Longtail butterfly ray	Indo–Pacific	Coastal and demersal	104	Near threatened	Ovoviviparity
*Himantura leoparda*	Leopard whipray	Indo–west Pacific	Coastal and demersal	140	Vulnerable	Ovoviviparous
*Himantura uarnak*	Honeycomb stingray/ Reticulate whipray	Indo–west Pacific Lessepsian migrant found in the Mediterranean Sea.	Coastal and intertidal	160	Vulnerable	Site affinity; ovoviviparous
*Maculabatis gerrardi*	Sharpnose stingray	Indo–west Pacific	Coastal and demersal	116	Endangered	Ovoviviparous
*Mobula birostris*	Giant oceanic manta ray	Indo-Pacific	Oceanic, coastal and demersal	670–900	Vulnerable	Site fidelity, seasonal residency; ovoviviparous
*Mobula kuhlii*	Shortfin devil ray	Indo–west Pacific	Coastal, oceanic and pelagic	135	Endangered	Ovoviviparous
*Mobula mobular*	Devil Fish	Global	Oceanic, coastal and pelagic	520	Endangered	Ovoviviparous
*Mobula tarapacana*	Sicklefin devil ray	Global	Coastal, oceanic and pelagic	370	Endangered	Ovoviviparous
*Mobula thurstoni*	Bentfin devil ray	Global	Coastal and pelagic	189	Endangered	Ovoviviparous
*Neotrygon indica*	Indian ocean blue spotted maskray	Western Indian Ocean	Coastal and reef-associated	31.4	Newly described species	
*Neotrygon kuhlii*	Bluespotted maskray	Southwest Pacific Ocean	Reef-associated and demersal	30	Data deficient	Site affinity, regional philopatry: ovoviviparous
*Pateobatis jenkinsii*	Jenskin’s whipray	Indo–Pacific	Coastal and demersal	150	Vulnerable	Ovoviviparous
*Pteroplatytrygon violacea*	Pelagic stingray	Global	Oceanic and pelagic	96	Least concern	Ovoviviparous
*Taeniura lymma*	Blue spotted ribbon ray	Indo–west Pacific	Coastal, reef-associated and benthic	30–35	Near threatened	Ovoviviparous

**Notes.**

a Disc Width refers to the maximum measured wing-span of the ray species.

b Traits refer to the type of philopatry and reproduction shown by the particular ray species.

### Alignment of sequences

The sequences were downloaded and aligned using ‘Clustal W’ in MEGA 5.05 (MEGA X: Molecular Evolutionary Genetics Analysis across computing platforms; Kumar et al., 2018). The length of shark sequences ranged from 648 bp to 687 bp and the sequence length for rays ranged from 651 bp to 693 bp. Stop codons, if present in the aligned DNA segment, were deleted and sequences re-aligned before constructing the haplotype networks.

### Data analysis

DNA Sequence Polymorphism 6.12.03 (DnaSP 6; Rozas et al., 2017) was used to generate the haplotype data file and calculate haplotype and nucleotide diversities (Nei, 1987; Nei & Miller, 1990) with corresponding standard deviations. The sample sites were categorised as Western, Eastern and Central Indian Ocean within the Indian Ocean group; Northern & Southern Atlantic within the Atlantic Ocean group; and Northern & Southern Pacific within the Pacific Ocean group. A Median Joining Haplotype Network (Bandelt, Forster & Röhl, 1999) was constructed using PopART 1.7 (Population Analysis with Reticulate Trees; Leigh & Bryant, 2015) for sharks (Table 1) and rays (Table 2). In population genetics, haplotype networks are used to understand biogeography and genealogical relationships at intraspecific levels (Leigh & Bryant, 2015). Median joining networks can handle large data sets (Posada & Crandall, 2001) and combines the features of minimum spanning trees (Kruskal’s algorithm) and Farris’s maximum-parsimony heuristic algorithm (Bandelt, Forster & Röhl, 1999).

All the observed haplotype networks were classified into seven types of topologies. Based on Jenkins, Castilho & Stevens (2018), we classified networks as star (a single dominant haplotype with many haplotypes related to it), complex mutational (a few haplotypes differing by one or two mutations and some by a very large number) and complex star (many dominant haplotypes and high frequency connections). When species from different geographic ranges did not share any haplotypes and differed by several mutations, we termed it as ‘simple exclusive’ (referred to as reciprocally monophyletic by some authors). We classified networks as ‘single’ when species from different geographic regions were represented by a single haplotype. The term ‘simple linear’ was used to refer to networks having three or more haplotypes arranged linearly with no branches. Networks that did not fall into any of the above mentioned six categories and had two or more haplotypes arranged in no particular pattern were categorized as ‘simple’.

Statistical analyses were carried out using PopART. To understand the extent of geographic structuring of evolutionary lineages, nested/hierarchical AMOVA (Excoffier, Smouse & Quattro, 1992) was conducted after defining the groups. The groups defined were Indian Ocean, Pacific Ocean, and Atlantic Ocean with subgroups as defined above. Fixation indices (Φ_ST_, Φ_SC_, Φ_CT_) and percentage variation among groups, among populations and within populations were reported from AMOVA. Differentiation among all populations *i.e.,* all subgroups is represented by overall Φ_ST_ values, while population structuring within individual ocean basins is represented by Φ_SC_; population structuring across ocean basins is given by Φ_CT_ values. Genetic structuring of populations between Indo–Pacific and Atlantic species was also carried out, wherein the samples from Indian and Pacific Oceans were combined (Indo–Pacific) and samples from Atlantic Ocean formed the second group. The extent of genetic structuring among shark and ray species from different regions was analysed using the haplotype networks, AMOVA, and diversity values.

Kruskal–Wallis (Siegel & Castellan, 1988) tests were carried out to statistically test if the haplotype (*h*) and nucleotide ( *π*) diversity values differed among different families of sharks and rays. The test was also carried out to determine if the diversity values differed significantly between species based on their habitat. A Mann–Whitney *U* test (Siegel & Castellan, 1988) was used to determine whether diversity values differed significantly between sharks and rays. Fisher’s exact test was used to assess the effect of structuring across habitats for both sharks and rays. The habitats occupied by species of both sharks and rays were classified into three broad categories–pelagic (includes semi pelagic species as well), demersal (includes benthopelagic species) and reef-associated (includes those that are bottom dwelling among coral reefs, *i.e.,* reef-associated and demersal).

## Results

Before presenting results, we reiterate that data was drawn primarily from published studies (for 11 shark and three ray species) but also include unpublished data (for 28 shark and 17 ray species; Tables S1 and S2). We regard the published data as highly reliable. The unpublished data was given a higher level of scrutiny, including contribution source, chronology, and volume of sequence data. In cases where unpublished data corroborated published data, we considered the results to be supported. In instances where observed divergent lineages were entirely based on unpublished data, we regarded the results as provisional. In particular, *Gymnura micrura* had highly divergent lineages and the results should be treated as provisional. The careful use of GenBank data, while provisional, is unlikely to introduce a systemic bias that would alter the interpretation of results.

### SHARKS: patterns of genetic structure and diversity

#### Network topology

**Figure 1:**
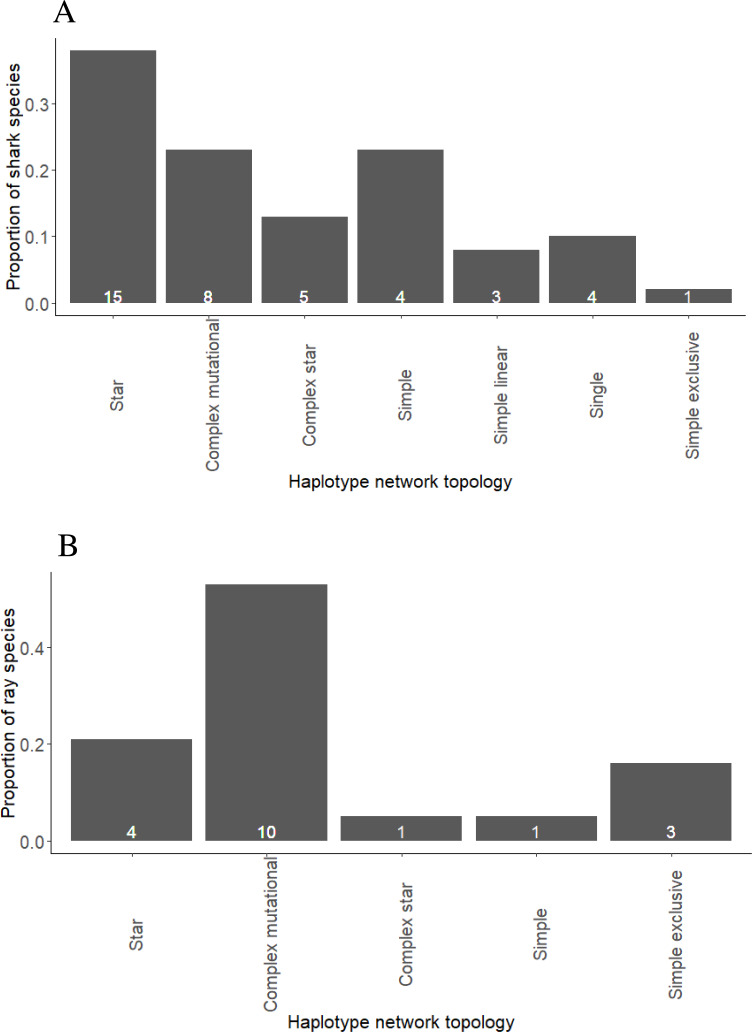
Proportion of shark (A) and ray (B) species exhibiting different haplotype network topologies. The total number of shark species in the present study is forty and number of ray species is nineteen.

Star networks were predominant among shark species, including eight pelagic, two reef-associated, two demersal and reef-associated, two demersal and one semi-pelagic species (Fig. 1A, Fig. S1 and Table S3). Complex mutational networks were observed in three reef-associated, one demersal & reef-associated, two semi-pelagic, one pelagic and one benthopelagic species. Many species exhibiting complex mutational networks also had a dominant (presumably) ancestral haplotype at the centre of a star topology. Complex star topologies were observed in four species inhabiting pelagic waters and one reef-associated species. The coastal-demersal *Ca. dussumieri* had two small clusters of haplotypes, corresponding to the Western Indian Ocean and Eastern Indian/South Pacific, separated by a long branch with 33 mutations (Fig. S1). Two globally distributed pelagic species (*P. glauca* and *Sp. zygaena*) and, one demersal (*Ch. Griseum*) and one Atlantic species (*N. brevirostris*) were represented by a single haplotype (Fig. S1 and Table S3).

#### Haplotype and nucleotide diversity

**Figure 2:**
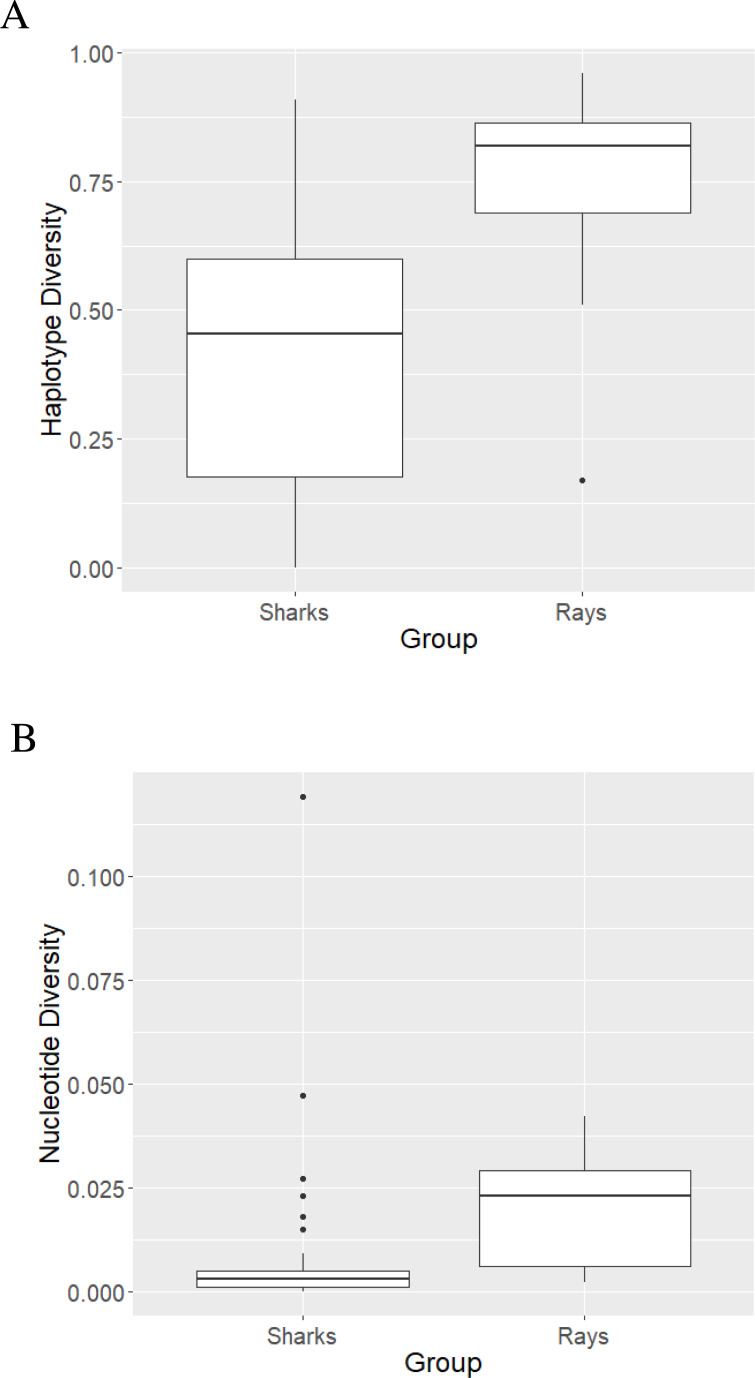
Haplotype (A) and nucleotide (B) diversity values of sharks and rays represented as boxplots.

The mean haplotype diversity value of sharks was 0.422 ±0.260 (Fig. 2A) with the highest diversity (*h* = 0.91 ± 0.047) observed in *L. ditropis*, a coastal-oceanic-pelagic species. The lowest (non-zero) diversity (*h* = 0.004 ± 0.066) was observed in *Carcharias carcharias* (Table S3). Within genus *Carcharhinus*, the haplotype diversity value was lowest for a coastal-demersal species *Ca. amboinensis* (*h* = 0.108 ± 0.049) and highest for *Ca. plumbeus*, a coastal-benthopelagic species (*h* = 0.671 ± 0.083). Haplotype diversity was high (*h* ≥ 0.50) for eighteen species, of which twelve were coastal. Haplotype diversity was low (*h* ≤ 0.50) for twenty-two species, with the majority (12) inhabiting oceanic/semi-oceanic waters (Table S3). There was no difference in *h* values between the nine families of sharks (Kruskal–Wallis *χ*^2^ = 738, *p* = 0.49).

The highest nucleotide diversity was observed in the coastal, reef associated *Rhiz. oligolinx* (*π* = 0.119 ± 0.00787) while the coastal-oceanic *Rhin. typus* had the lowest (non-zero) diversity (0.0002 ± 0.0001; Table S3). The mean nucleotide diversity value for sharks was 0.0086 ± 0.02 (Fig. 2B). Within genus *Carcharhinus*, coastal, reef associated *Ca. dussumieri* had the highest nucleotide diversity (*π* = 0.027 ± 0.0039) while oceanic/pelagic *Ca. longimanus* had the lowest (*π* = 0.0003 ± 0.0001). The differences in nucleotide diversity for the remaining eight species within the genus *Carcharhinus* were minimal. High nucleotide diversity values (*π* ≥ 0.005) were observed in twelve species with the most common habitat being pelagic waters (five). Twenty-eight species had low nucleotide diversity values (*π* ≥ 0.005) with the most common habitat being pelagic (10) followed by reef-associated (six) and demersal (five). There was no difference in *π* values between the nine families of sharks (Kruskal–Wallis *χ*^2^ = 6.55, *p* = 0.59). There was no difference in diversity values between species occupying different habitats—*h* (*χ*^2^ = 4.04, *p* = 0.40) and *π* (*χ*^2^ = 2.78, *p* = 0.59).

#### Genetic structure

**Figure 3:**
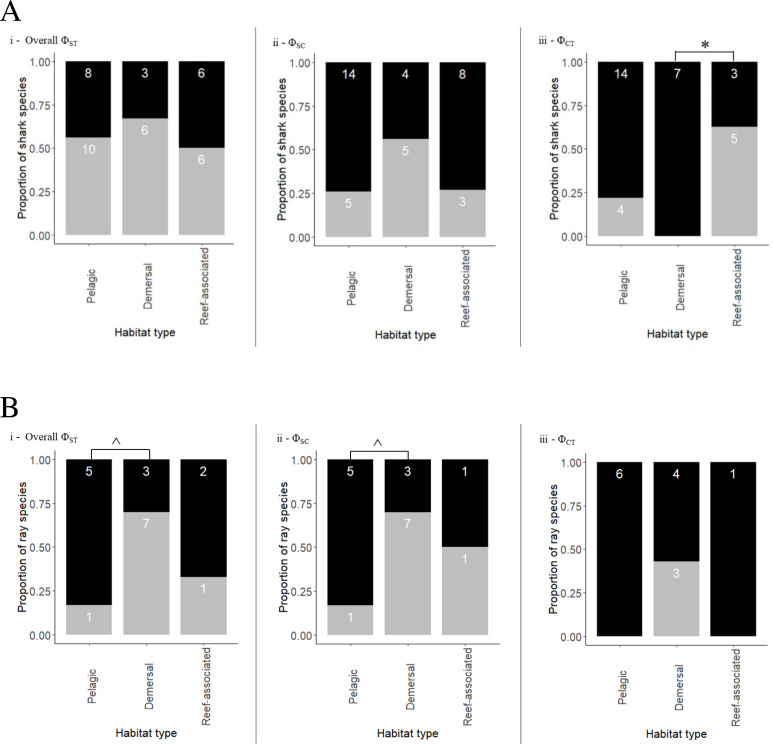
Proportion of shark (A) and ray (B) species exhibiting population structuring/no structuring in relation to their habitats across different Φ statistics (grey bars represent significant structuring; black bars represent no structure). (i) Population structuring among all populations (overall Φ_*ST*_), (ii) population structuring within ocean basins (Φ_*SC*_), (iii) population structuring across ocean basins (Φ_*CT*_). Semi pelagic species have been grouped with pelagic species and benthopelagic with demersal species. Demersal & reef-associated species have been grouped with reef-associated species. Asterisk (*) indicates the *p*-value (*p* = 0.026) of test of proportions. Caret (^∧^) indicates the *p*-value (*p* = 0.13) for the test of proportions.

The coastal-demersal *Ca. dussumieri* had the highest overall Φ_ST_ value (0.99, *p* < 0.001; Table 3). Eleven of 22 species (50%) belonging to the family Carcharhinidae and four pelagic species of family Lamnidae (80%) had significant overall Φ_ST_ values. Most species that exhibited structuring within Carcharhinidae were reef-associated and/or demersal. *Isurus oxyrinchus* had the lowest Φ_ST_ value (0.067, *p* = 0.018) indicating weak but significant structuring. Very weak structuring was observed in *Ca. longimanus*, *I. oxyrhincus* and *G. cuvier*, all inhabiting pelagic and semi pelagic waters. Four pelagic and one semi pelagic species with Indo–Pacific/global distribution showed negative or Φ_ST_ = 0 values, indicating a lack of structure among oceans. Overall, there was no significant difference between habitats in Φ_ST_ (Fisher’s exact test, *p* = 0.796) (Fig. 3A; See Table 3 for a summary).

Nested AMOVA results show significant structuring of population samples among oceans (Φ_CT_) in five reef-associated, one semi pelagic and three pelagic species (Fig. 3A; Table 3). Among these, four reef-associated, one semi-pelagic and two pelagic species had distribution ranges in the Indo–Pacific, indicating that structuring was primarily between the Indian and Pacific Ocean. In contrast to the findings above for Φ_ST_, Φ_CT_ was significant (Fisher’s exact test *p* = 0.018) for species occupying different habitats. Pairwise comparison revealed that the proportion of structured populations was significantly lower in demersal species than reef-associated species (*p* = 0.026).

**Figure 4:**
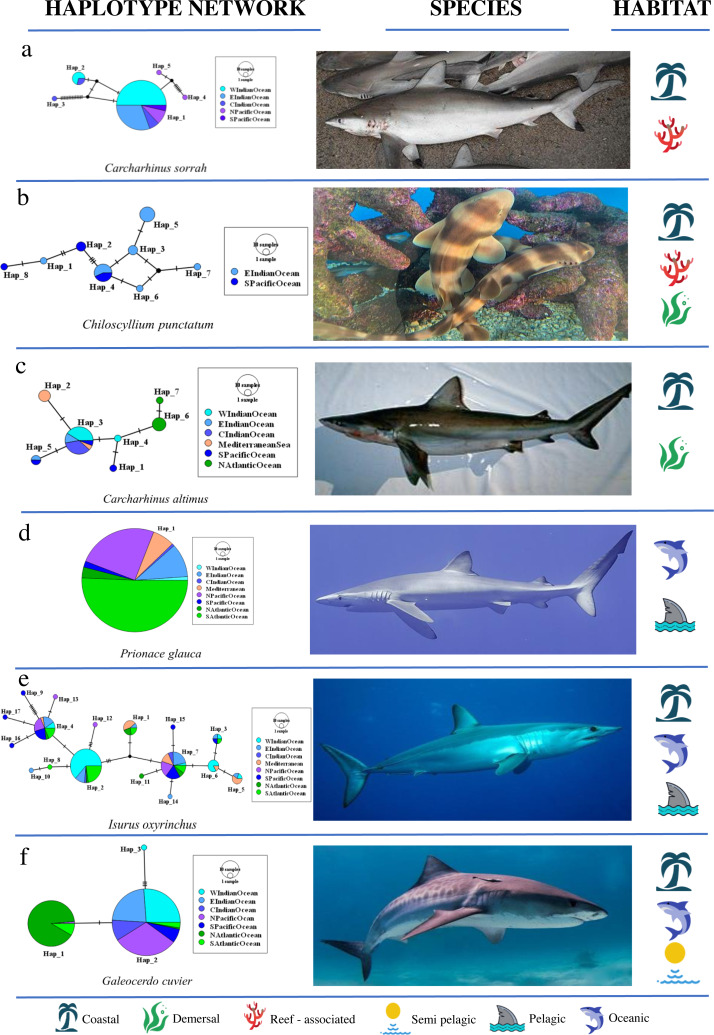
Haplotype network structures of six shark species representing different habitats. All images have been taken from Wikimedia Commons. Picture credits: (A) *Carcharhinus sorrah*, Tassapon Krajangdara, CC BY 3.0; (B) *Chiloscyllium punctatum*, Coughdrop12, CC BY-SA 4.0; (C) *Carcharhinus altimus*, NOAA; (D) *Prionace glauca,* Diego Delso, CC BY-SA 4.0; (E) *Isurus oxyrinchus*, Patrick Doll, CC BY-SA 3.0; (F) *Galeocerdo cuvier*, Albert Kok, CC BY-SA 3.0. The icons representing habitat type are available at https://icons8.com.

*Ca. macloti* a reef-associated & demersal species had the highest Φ_CT_ value (0.894, *p* < 0.001) while the lowest was observed in two reef-associated species (*Ca. sorrah* and *Ca. limbatus*). Pelagic and semi-pelagic species in five families had non-significant Φ_CT_ values, thereby lacking structuring across the three (or two Indian & Pacific) ocean basins. A few reef-associated, demersal and three benthopelagic sharks also lacked structuring (Fig. 3A). Differentiation among population samples within ocean (Φ_SC_) was significant for 13 species. These are five reef-associated and/or demersal, three benthopelagic, three pelagic and two semi-pelagic species (Fig. 3A; Table 3). *Carcharias taurus* had the highest Φ_SC_ value (0.994, *p* < 0.001; reef-associated & demersal) and the lowest was for *I. oxyrinchus* (0.112, *p* = 0.016; oceanic & semi pelagic). There was no significant difference in the proportion of structured populations within ocean basins (Fisher’s exact test, *p* = 0.37) across the three habitats. In comparisons of Indo–Pacific *vs* Atlantic groupings, three of 17 globally distributed species (*Ca. longimanus*, *G. cuvier* and *I. paucus*) showed structuring. *A. superciliosus*, *Ca. brevipinna*, *Ca. amblyrhynchoides*, *Ca. amboinensis*, *P. glauca*, *Ce. maximus*, *Rhin. typus*, *Sp. mokarran* and *Sp. zygaena* lacked detectable structuring at all levels (Table 3). A few haplotypes of *Ca. brevipinna* are separated by more mutations from the central haplotype compared to *Ca. amboinensis*, in the same region (Indo-Pacific). This could be due to differences in the number of sequences used covering a broader range for *Ca. brevipinna* (132 sequences), while *Ca. amboinensis* was represented by 72 sequences. See Fig. 4A for an overview of the network topologies and life history characteristics of five shark species.

**Table 3:** AMOVA analysis carried out using cytochrome C oxidase subunit I for sharks. The numerals in bold indicate significant structuring, *p* values are given in brackets and *n* is the sample size.

**Species name**	Overall Φ_**ST**_	**Global comparison (nested AMOVA)**			**Indo–Pacific vs Atlantic**		
		Φ_**ST**_	Φ_**SC**_	Φ_**CT**_	Φ_**ST**_	Φ_**SC**_	Φ_**CT**_
*A. pelagicus* (*n* = 146)	0.211^**^	0.308^**^	0.218^*^ **(0.002)**	0.116^**^	Indo–Pacific distribution		
*A. superciliosus* (*n* = 104)	−0.07 (0.882)	−0.066 (0.755)	−0.149 (0.949)	0.072 (0.228)	−0.12 (0.735)	−0.078 (0.814)	−0.038 (0.793)
*A. vulpinus* (*n* = 44)	0.513^**^ **(0.001)**	0.613^**^	0.374 (0.365)	0.382^*^ **(0.025)**	0.56^**^ **(0.001)**	0.59^*^ **(0.019)**	−0.086 (0.267)
*Ca. altimus* (*n* = 29)	0.714^**^	0.735^**^	0.787^**^	−0.245 (0.602)	0.759^**^	0.75^*^ **(0.003)**	0.038 (0.297)
*Ca. amblyrhynchoides* (*n* = 32)	0.024 (0.296)	−0.105 (0.875)	0.303 (0.144)	0.152 (0.728)	Indo–west Pacific distribution		
*Ca. amboinensis* (*n* = 72)	0.018 (0.601)	−0.086 (0.904)	0.065 (0.246)	0.162 (0.888)	Indo–Pacific distribution		
*Ca. brevipinna* (*n* = 132)	0.052 (0.183)	0.036 (0.423)	0.124 (0.06)	0.106 (0.807)	0.0018 (0.537)	0.11 (0.222)	−0.13 (0.527)
*Ca. dussumieri* (*n* = 24)	0.99^**^	0.99^**^	0.99^**^	−0.388 (0.665)	Indo–Pacific distribution		
*Ca. falciformis* (*n* = 116)		0.206 (0.081)	−0.054 (0.483)	0.247^*^ **(0.007)**			
*Ca. leucas* (*n* = 66)	0.37 (0.017)	0.423^*^ **(0.003)**	0.398 (0.046)	0.041 (0.279)	0.369^*^ **(0.015)**	0.46^*^ **(0.029)**	−0.17 (0.477)
*Ca. limbatus* (*n* = 78)	0.244^*^ **(0.008)**	0.238^*^ **(0.02)**	0.007 (0.22)	0.067^**^	0.22^*^ **(0.046)**	0.35^*^ **(0.016)**	−0.201 (0.915)
*Ca. longimanus* (*n* = 30)	0.091^*^ **(0.058)**	0.163 (0.15)	−0.289 (0.497)	0.351 (0.096)	0.306^*^ (0.032)	−0.175 (0.17)	0.409^**^
*Ca. macloti* (*n* = 12)	0.633^**^	0.915^**^	0.191^**^	0.894^**^	IP	IP	IP
*Ca. melanopterus* (*n* = 54)	0.408^*^ (0.021)	0.735^**^	0.151 (0.291)	0.692^*^	Indo–Pacific distribution		
*Ca. plumbeus* (*n* = 48)	0.493^*^	0.601^**^	0.645^**^	−0.122 (0.087)	0.582^**^	0.649^**^	−0.109 (0.791)
*Ca. sealei* (*n* = 18)	−0.013 (0.292)	All sequences from Indian Ocean					
*Ca. sorrah* (*n* = 124)	0.012 (0.521)	0.06 (0.345)	0.007 (0.601)	0.067^**^	Indo–Pacific distribution		
*Ca. taurus*	0.997^**^	0.998^**^	0.994^**^	0.667 (0.105)	0.998^**^	0.993^**^	0.756^*^ (0.05)
*Carcharodon carcharias* (*n* = 18)	0.379^*^ (0.041)	0.442^*^ (0.026)	0.355 (0.361)	0.134 (0.187)	0.342 (0.084)	0.501^*^ (0.027)	−0.319 (0.879)
*Ce. maximus* (*n* = 56)	−0.0104 (0.246)	0.0005 (0.2250)	−0.065 (0.329)	0.066 (0.389)	−0.013 (0.3)	−0.022 (0.31)	0.009 (0.478)
*Ch. griseum* (*n* = 12)	All sequences from central Indian Ocean						
*Ch. indicum* (*n* = 14)	All sequences from eastern Indian Ocean						
*Ch. punctatum* (*n* = 20)	0.206^*^ (0.019)	All sequences from eastern Indian and south Pacific Ocean					
*G. cuvier* (*n* = 228)	0.068^**^	0.833^**^	0.174^*^ (0.006)	0.798^*^ (0.057)	0.88^**^	0.13^*^ (0.009)	0.86^**^
*I. oxyrinchus* (*n* = 140)	0.067^*^	0.0707^*^ (0.037)	0.112^*^ (0.016)	−0.047 (0.698)	0.085^*^	0.076^*^	0.0104
*I. paucus* (*n* = 46)	0.401^**^	0.519^**^	0.157 (0.163)	0.429 (0.123)	0.562^**^	0.107 (0.213)	0.509^**^
*L. ditropis* (*n* = 15)	North Pacific distribution						
*L. nasus* (*n* = 81)	0.455^**^	0.427^**^	0.646^**^	−0.621 (0.731)	All sequences from Atlantic and Pacific Ocean		
*N. acutidens* (*n* = 31)	0.568^**^	0.718^**^	0.714^**^	0.016 (0.325)	Indo–Pacific distribution		
*N. brevirostris* (*n* = 11)	0	Atlantic distribution					
*P. glauca* (*n* = 534)	0	0	0	0	0	0	0
*Rhin. typus* (*n* = 48)	−0.001 (0.935)	−0.055 (0.993)	−0.05 (0.532)	0.0022 (0.482)	All sequences from Indo–Pacific		
*Rhiz. acutus* (*n* = 78)	0.678^**^	0.818^**^	0.744^**^	0.286 (0.263)	Indo–west Pacific distribution		
*Rhiz. oligolinx* (*n* = 15)	0.026 (0.37)	All sequences from Atlantic Ocean					
*S. laticaudus* (*n* = 27)	−0.0105 (0.622)	All sequences from Indian Ocean					
*Sp. mokarran* (*n* = 59)	0.084 (0.129)	0.075 (0.153)	0.361 (0.0156)	−0.447 (0.728)	0.095 (0.139)	0.24 (0.202)	−0.188 (0.704)
*Sp. lewini* (*n* = 323)	0.726^**^	0.80^**^	0.76^**^	0.207 (0.344)	0.734^**^	0.81^**^	−0.375 (0.678)
*Sp. zygaena* (*n* = 91)	0	0	0	0	0	0	0
*St. fasciatum* (*n* = 26)	0.306^*^ (0.013)	0.28^*^ (0.071)	0.466^*^ (0.017)	−0.35 (0.495)	Indo–Pacific distribution		
*T. obesus* (*n* = 53)	0.467^**^	0.654^**^	0.843^*^ (0.05)	−1.2 (0.767)	All sequences from Indo–Pacific		

**Notes.**

* *p* < 0.05.

** *p* < 0.001.

### RAYS: patterns of genetic structure and diversity

#### Network topology

Complex mutational topologies were observed in 10 out of 19 ray species (Fig. 1B, and Fig. S2 and Table S4), including one pelagic, two benthopelagic, two reef-associated & demersal, four demersal, one reef-associated species. Long branches between two clusters of haplotypes were observed in three demersal species while complex star topology was observed in one pelagic species. Star shaped haplotype networks were observed in only four species of rays (Fig. S2, Table S4) inhabiting pelagic (three) and benthopelagic (one) habitats.

#### Haplotype and nucleotide diversity

The mean haplotype diversity value was 0.74 ± 0.217 (Fig. 2A). *B. walga*, a coastal-benthic species, had the highest haplotype diversity (*h* = 0.963 ± 0.028) among rays while the pelagic *Mob. tarapacana* had the lowest (*h* = 0.17 ± 0.021; Table S4). All the species belonging to family Dasyatidae had high *h* values (0.67 to 0.91) while the diversity values for family Mobulidae ranged from 0.17 to 0.85. Haplotype diversity was high (*h* ≥ 0.50) for eighteen species representing all habitats, while only one pelagic species—*Mob. tarpacana* had a low value (*h* = 0.17 ± 0.102). The mean nucleotide diversity value for rays was 0.0184 ± 0.0134 (Fig. 2B). Nucleotide diversity was highest in the coastal-demersal *G. poecilura* (*π* = 0.042 ± 0.0087) while *Mob. birostris* and *Mob. tarapacana* had the lowest value (*π* = 0.002; Table S4). In rays, all the nucleotide diversity values were lower than 0.005 (Table S4). There were no differences in the diversity values between ten families of rays, *h* (Kruskal–Wallis *χ*^2^ = 12.19, *p* = 0.25) and *π* (*χ*^2^ = 15.86, *p* = 0.069). However, demersal habitats were significantly higher in *h* and *π* relative to pelagic habitats (for *h, p* = 0.03 and for *π*, *p* = 0.002). The mean of both the diversity values was significantly higher for rays than for sharks—*h* (Mann Whitney *U* test, *p* < 0.0000096) and *π* (Mann Whitney *U* test, *p* < 0.00016).

#### Genetic structure

*Gymnura micrura* (demersal) showed very high structuring (Φ_ST_ = 0.989, *p* < 0.001) between North and South Atlantic samples with no sharing of haplotypes (simple exclusive topology; Table 3). *A. narinari* showed weak but significant structuring between the North and South Atlantic Ocean. The Western and Central Indian Ocean samples of *N. indica* (reef-associated) differed significantly with no haplotype sharing (Φ_ST_ = 0.357, *p* < 0.001). *Himantura leoparda* and *G. poecilura* showed highly significant population structure (Φ_ST_ = 0.918, *p* < 0.001 and 0.989, *p* < 0.001 respectively). In addition to these cases, three globally distributed species and four Indo–Pacific species had significant overall Φ_ST_values. No significant structuring was observed in ten species, including *T. lymma*, *B. imbricata*, *N. kuhlii*, *Pa. jenkinsii, Pt. violacea*, *Mob. kuhlii*, *Mob. tarapacana, Mob. thurstoni*, *Mob. birostris*, and *A. ocellatus* (Fisher’s exact test, *p* = 0.13). Of these, five are pelagic, two are reef-associated & demersal, one is benthopelagic and two are demersal species (Fig. 3B). (See Table 4 for a summary).

**Table 4:** AMOVA analysis carried out using cytochrome C oxidase subunit I for rays. The numerals in bold indicate significant structuring, *p* values are given in brackets and *n* is the sample size.

**Species name**	**Simple Φ**_**ST**_	**Global comparison (nested AMOVA)**			**Indo–Pacific vs Atlantic**		
		Φ_**ST**_	Φ_**SC**_	Φ_**CT**_	Φ_**ST**_	Φ_**SC**_	Φ_**CT**_
*A. narinari* (*n* = 29)	0.021^*^ (0.007)	Atlantic Ocean distribution					
*A. ocellatus* (*n* = 18)	**-**0.028 (0.522)	−0.12 (0.704)	0.117 (0.218)	0.27 (0.674)	Indo–Pacific distribution		
*B. imbricata* (*n* = 23)	0.011 (0.317)	All sequences from western or central Indian Ocean					
*B. walga* (*n* = 20)	0.567^*^ (0.002)	0.688^**^	0.714^**^	0.092 (0.327)	Indo–Pacific distribution		
*G. micrura* (*n* = 15)	0.989^**^	All sequences from north or south Atlantic					
*G. poecilura* (*n* = 39)	0.339^**^ (0.001)	0.438^**^ (0.001)	0.618^*^ (0.026)	0.618 (0.247)	Indo–Pacific distribution		
*H. leoparda* (*n* = 23)	0.918^**^	0.974^**^	0.626^*^ (0.025)	0.931^**^	Indo–Pacific distribution		
*H. uarnak* (*n* = 49)	0.636^**^	0.915^**^ (0.001)	0.913^**^	0.019 (0.359)	Indo–Mediterranean distribution		
*Mac. gerrardi* (*n* = 37)	0.318^**^ (0.001)	0.595^*^ (0.015)	0.384^*^ (0.004)	0.344^**^	Indo–Pacific distribution		
*Mob. birostris* (*n* = 16)	0.05 (0.364)	−1.09 (0.543)	0.165 (0.116)	−1.502 (0.751)	Indo–Pacific distribution		
*Mob. kuhlii* (*n* = 18)	−0.278 (0.998)	−0.462 (0.96)	−0.457 (0.999)	−0.0032 (0.826)	Indo–Pacific distribution		
*Mob. mobular* (*n* = 15)	0.1587^*^ (0.044)	0.228^*^ (0.052)	0.165^*^ (0.013)	0.774 (0.145)	0.277 (0.135)	0.208^**^	0.087 (0.213)
*Mob. tarapacana* (*n* = 23)	−0.062 (0.659)	−0.134 (0.905)	−0.0086 (0.534)	−0.124 (0.682)	−1.057 (0.642)	−0.0267 (0.432)	−1.0043 (0.772)
*Mob. thurstoni* (*n* = 32)	0.099 (0.116)	0.201 (0.085)	−0.1014 (0.409)	0.274 (0.28)	0.341 (0.059)	0.053 (0.328)	0.304 (0.301)
*N. indica* (*n* = 15)	0.357^**^	All sequences from western or central Indian Ocean					
*N. kuhlii* (*n* = 59)	0	Southwest Pacific Ocean distribution					
*P. jenkinsii* (*n* = 22)	0.074 (0.272)	All sequences from central or eastern Indian Ocean					
*P. violacea* (*n* = 25)	−0.812 (0.782)	−0.233 (0.628)	−0.291 (0.887)	0.045 (0.683)	−0.204 (0.666)	−0.332 (0.859)	0.096 (0.31)S
*T. lymma* (*n* = 20)	−0.046 (0.671)	Indo–Pacific distribution					

**Notes.**

* *p* < 0.05.

** *p* < 0.001.

**Figure 5:**
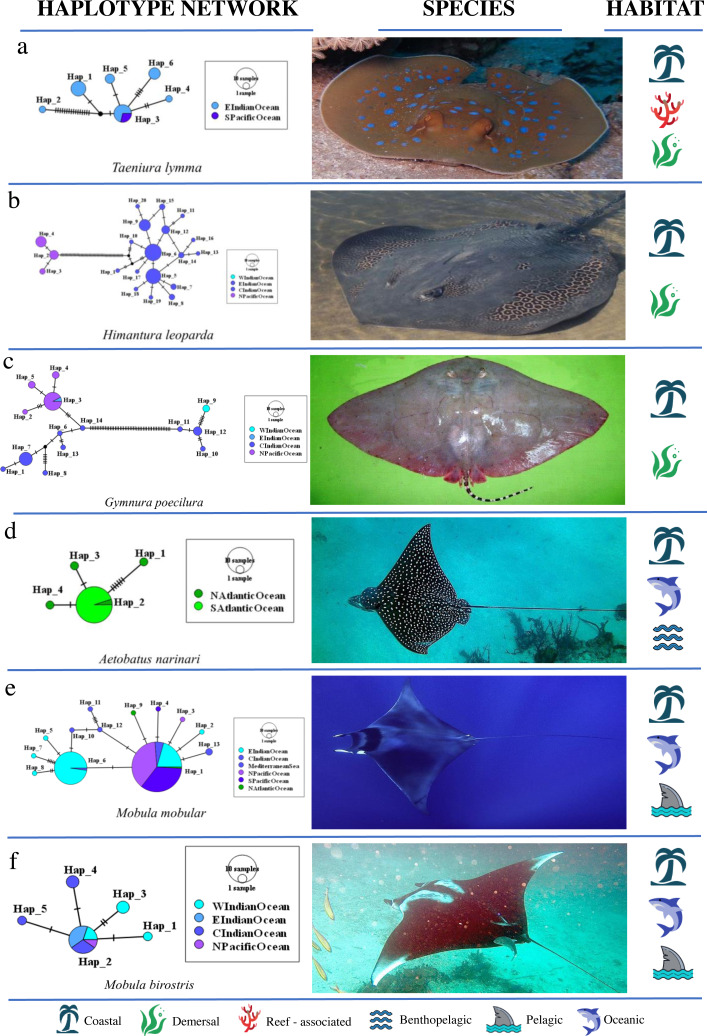
Haplotype network structures of six ray species representing different habitats. All images have been taken from Wikimedia Commons. Picture credits: (A) *Taeniura lymma*, Jon Hanson CC BY-SA 2.0; (B) *Himantura leoparda*, Julie Lawson ; (C) *Gymnura poecilura*, Hamid Badar CC BY 3.0; (D) *Aetobatus narinari*, Nicholas Lindell Reynolds CC BY-SA 4.0; (E) *Mobula mobular*, Julien Renoult ; (F) *Mobula birostris*, Jon Hanson CC BY-SA 2.0. The icons representing habitat type are available at https://icons8.com/ (Icons8 LLC., 2023).

A nested AMOVA revealed that none of the four globally distributed species showed structuring across the three major ocean basins. Two of nine Indo–Pacific species (*H. leoparda* and *Mac. gerrardi*) showed structuring (Table 4). Structuring at the level of ocean basins was only detected in benthopelagic and demersal rays (Fig. 3B). There was no significant difference in the proportion of structured population samples among oceans (Fisher’s exact test, *p* = 0.25). Variation among populations within an ocean basin (Φ_SC_) was significant for nine species of rays–six demersal, one benthopelagic and two pelagic (Fig. 3B). The highest variation within oceans was observed in *G. micrura* (Φ_SC_ = 0.989, *p* = 0.001, demersal) followed by an intertidal species–*H. uarnak* (Φ_SC_ = 0.913, *p* < 0.001; Lessepsian migrant), found in Indian Ocean and Mediterranean Sea (Table 4). There was no significant difference in the proportion of structured and non-structured population samples within ocean basins (Fisher’s exact test, *p* = 0.13). None of the four globally distributed ray species showed Indo–Pacific *vs* Atlantic structuring. Seven species of rays having distribution ranges in either all three or two ocean basins lacked structuring at all levels–five pelagic (four belong to genus *Mobula*), one reef-associated (*T. lymma*) and one demersal (*M. birostris*) species. See Fig. 5 for an overview of the network topologies and life history characteristics of five ray species.

## Discussion

### Genetic structure in sharks and rays

There are at least three tiers of population structure in marine fishes. The low-dispersal damselfishes and anemonefishes (family Pomacentridae) can have population structure at the scale of individual bays and archipelagos (*e.g.*, Dohna et al., 2015; Tenggardjaja, Bowen & Bernardi, 2016). This is often attributed, at least in part, to a greatly attenuated pelagic larval duration (PLD), but larval duration can only provide part of the answer. In a survey of 35 reef-associated species across the Hawaiian Archipelago, Selkoe et al. (2014) could attribute only 50% of the variance in population structure to PLD. The second tier is coastal fishes with broad ranges in the Indo–Pacific and Atlantic. These species typically have population structures at the scale of biogeographic provinces such as the Caribbean *vs* Brazil (Rocha et al., 2002), Red Sea *vs* Western Indian Ocean (Coleman et al., 2016), or Hawaiian Archipelago *vs* West Pacific (Leray et al., 2010). The third tier is pelagic wanderers or those that have very long PLD, including tunas (family Scombridae, Pecoraro et al., 2018; Puncher et al., 2018; Rodríguez-Ezpeleta et al., 2019) and billfishes (family Istiophoridae, Graves & McDowell, 2015). These species show population structure at the scale of ocean basins. Some moray eels (family Muraenidae), with PLDs exceeding 100 days, show no population structure across the entire Indo–Pacific basin (Reece, Bowen & Larson, 2011). Deepwater species, those below 200 m depth, seem to fall into this category as well (Andrews et al., 2020; McDowell & Brightman, 2018).

Based on our analysis of existing data sets, sharks and rays (lacking a PLD) fall primarily in the second tier (biogeographic provinces) and third tier (ocean basins) because a majority of the genetic structuring was observed within or across ocean basins. Examples of the second tier include pelagic sand tiger shark (*Carcharias taurus*; Ahonen, Harcourt & Stow, 2009), and blacktip reef shark (*Ca. melanopterus*; Vignaud et al., 2014) where structuring was observed within the Atlantic and Pacific oceans respectively. The genetic structure within the Atlantic basin (Northwest Atlantic and Brazil samples) for sand tiger sharks has been attributed to the warm equatorial currents acting as a barrier along with high breeding site fidelity (Dicken et al., 2007; Ahonen, Harcourt & Stow, 2009; Bansemer & Bennett, 2009), while for blacktip reef sharks, philopatry and deep oceanic waters influence genetic structuring (Vignaud et al., 2014). Shortfin mako shark (*I. oxyrhynchus*; Heist, Musick & Graves, 1996) and zebra shark (*St. fasciatum*; Dudgeon, Broderick & Ovenden, 2009) exhibited structuring at the level of biogeographic provinces. The porbeagle shark (*L. nasus*) has an anti-tropical distribution (absent in tropical waters) because it prefers regions where the mean water temperature is 7–18 °C (González et al., 2021). In the present study, South Atlantic and South Pacific samples comprise a southern lineage, and samples from the Mediterranean Sea and North Atlantic are united in a northern lineage. *Isurus oxyrinchus*, a highly migratory species also shows anti-tropical structuring. It is known to exhibit extended periods of residency and has an affinity to coastal waters and therefore does not frequently undertake trans-equatorial migrations (Corrigan et al., 2018). Apart from the ones mentioned above, bignose shark (*Ca. altimus*), sandbar shark (*Ca. plumbeus*), lemon shark (*N. acutidens*), milk shark (*Rhiz. acutus*), scalloped hammerhead shark (*S. lewini*) and whitetip reef shark (*T. obesus*) showed structuring within basins. Among rays, the spotted eagle ray (*A. narinari*), scaly whipray (*B. walga*), reticulate whipray (*H. uarnak*), blue spotted stingray (*N. indica*), giant devil ray (*Mob. mobular*), longtail butterfly ray (*G. poecilura*) and smooth butterfly ray (*G. micrura*) showed within basin structuring.

In the third tier, several oceanic and pelagic sharks exhibit population structure between the Atlantic and Indo–Pacific basins. These include the whale shark (*Rhin. typus*) (Φ_ST_ = 0.107; Castro et al., 2007), longfin mako (*I. paucus*; present study) and oceanic whitetip shark (*Ca. longimanus*; present study). This is a common pattern in pelagic teleost fishes where there is genetic structuring between the Atlantic and Indo–Pacific with little to no genetic structuring within ocean basins (Graves & McDowell, 2015; Bowen et al., 2016). At least two pelagic sharks show low-to-no genetic structure worldwide: basking shark (*Ce. maximus*; Hoelzel et al., 2006), and blue shark (*P. glauca*; Veríssimo et al., 2017). Other pelagic sharks with more limited distribution–bigeye thresher shark (*A. superciliosus*), great hammerhead shark (*Sp. mokarran)*, smooth hammerhead shark (*Sp. zygaena*) and spinner shark (*Ca. brevipinna*)–showed no structuring (panmictic population) across their entire range. Silky shark (*Ca. falciformis*; pelagic) and spot-tail shark (*Ca. sorrah*) showed structured lineages only between three and two (Indo–Pacific) basins respectively.

In rays, such clear structuring was not observed wherein the species showed genetic separations only between ocean basins. This difference can be attributed to the habitat preference of target species, with the majority of the ray species being demersal and exhibiting genetic structuring within ocean basins. While pelagic rays lacked genetic structuring at all levels, two demersal species (*H. leoparda* and *Mac. gerrardi*) had structuring both within and between ocean basins. Pelagic stingray (*P. violacea*), and five pelagic species—*Mob. kuhlii*, *Mob. tarapcana*, *Mob. thurstoni*, *Mob. birostris* and *A. ocellatus* showed no structuring, possibly indicating greater gene flow between and within ocean basins.

The mean haplotype and nucleotide diversity (*h* and *π*) of rays were significantly higher than that of sharks. While sharks did not show any significant differences in diversity among species occupying different habitats, rays showed significantly higher diversity in species occupying demersal (rather than pelagic) habitats. This is in keeping with the observation that pelagic organisms are more dispersive and have geographically larger populations. However, neither sharks nor rays showed significant differences in diversity values among families.

### Drivers of geographical genetic structure

Habitat and depth preference clearly shapes the geographical genetic structure of sharks and rays (Hirschfeld et al., 2021; Canfield, Galván-Magaña & Bowen, 2022). Among sharks, genetic structure between ocean basins was observed predominantly in the shallow reef-associated species, but four pelagic (one of which is semi pelagic) sharks also showed this type of structuring. In both sharks and rays, within basin structuring was observed primarily in benthopelagic and reef-associated and/or demersal species, although exceptions did exist where pelagic species also showed structuring. In rays, structure of lineages was observed primarily in demersal and benthopelagic species (categorised as demersal in Fig. 3B). A few reef-associated and pelagic sharks and rays also showed within ocean basin structuring. There was a significant difference in the proportion of structured species between demersal and reef-associated sharks across ocean basins but not within basins.

While one semi-pelagic and three pelagic sharks showed genetic structure at the scale of Indo–Pacific *vs* Atlantic, none of the ray species exhibited this structuring. However, this comparison has limited utility as most rays had distributions limited to the Indo–Pacific (8), with one species (*H. uarnak*) found in Indian Ocean and Mediterranean Sea (Table 2). *H. uarnak*, with a natural distributional range in the Indo–Pacific, is the largest Lessepsian elasmobranch species reported from the Mediterranean Sea (Golani, 1998; Ali et al., 2010; Amor et al., 2016). This species showed within basin structuring. A significant difference in the proportion of structured species at the ocean basin level (Φ_CT_), was observed between demersal and reef-associated shark species but not in rays. The test of proportions for structuring between demersal and pelagic rays for overall Φ_ST_ and Φ_SC_ was not significant when all three habitats were considered. However, there appeared to be a stronger trend when reef-associated species were excluded from the analysis - Φ_ST_ and Φ_SC_ (Fisher’s exact test, *p* = 0.13). Therefore, the inability to observe significant differences between species of these two habitats in the present study may be due to small sample sizes.

The dispersal of shark and ray species is entirely mediated by the active movement of juveniles and adults, unlike teleosts whose dispersal depends on planktonic larval stage as well as oceanic circulation (Taguchi et al., 2015). As expected, large-bodied oceanic sharks tend to have a lower population structure (Hirschfeld et al., 2021). Adult mediated population connectivity (AMPC) may result in different population structuring because the ability to overcome physical-biological barriers will be different across ontogenic stages (Frisk, Jordaan & Miller, 2014). Greater genetic connectivity may be observed in AMPC when compared to the classical larval-mediated geneflow as genetic exchange occurs over large distances—100s to 1,000s km (Frisk et al., 2010; Frisk, Jordaan & Miller, 2014). In winter skates (*Leucoraja ocellata*), adult migration strongly influenced connectivity and was responsible for increased abundance of the species along George’s Bank (Frisk et al., 2010). Apart from causing an increase in the abundance of species during a particular season, adult migrations also result in open populations where emigration and immigration play important roles in maintaining connectivity among locations (Frisk et al., 2010).

Just like in any landscape, physical barriers in the marine environment affect the movement of individuals. The three major ocean basins are separated by the Isthmus of Panama, Old World Barrier and the Sunda Shelf Barrier (also referred to as Indo–Pacific barrier). Ocean basins also have mid-oceanic barriers like the East Pacific Barrier, Indian Ocean Barrier and Mid-Atlantic Barrier. Thermal barriers (equatorial warm-water barrier and Aghulas-Benguela), ocean currents, hyaline barriers, straits and depth also hinder the mobility of marine organisms (Toonen et al., 2016; Hirschfeld et al., 2021; Canfield, Galván-Magaña & Bowen, 2022). These physical and environmental barriers pose different constraints on species with varying life histories and would therefore influence the genetic structuring of sharks and rays differently. Apart from the limitations imposed by geophysical barriers, wide-ranging pelagic species may exhibit population structuring due to philopatry.

Philopatric behaviour has been documented in a variety of marine taxa including at least 31 sharks (Chapman et al., 2015). Every species has unique migrational tendencies and reproductive strategies which guide their movement; therefore, it is not possible to find a general pattern of stock structure or gene flow that would apply to all species, even those occupying similar habitats (Heist, 2008). For example, tiger sharks (*G. cuvier*) have a regional population structure even though they undertake trans-oceanic migrations (Ferreira et al., 2015). This population genetic structure, detected in the maternally-inherited mtDNA, is attributed to female site fidelity (philopatry) to reproductive areas (Bernard et al., 2016), resulting in more structured populations (Chapman et al., 2004). Tiger sharks are monandrous and polyandry has not been detected in this species (Pirog et al., 2020). On the other hand, population structuring in pelagic thresher sharks and silky sharks may be shaped by oceanic currents and geography (Cardeñosa, Hyde & Caballero, 2014; Clarke et al., 2015; Domingues et al., 2018; Kraft et al., 2020). Juvenile sharks have been observed to remain in their natal sites for a long time before moving to habitats used by older juveniles and then to those used by adults (Springer, 1967). Hueter et al. (2005) reported that the traits such as residency, site fidelity and philopatry, either in combination or alone, influence population structuring at finer geographic scales among coastal shark species. Therefore, behavioural patterns (like philopatry) that inhibit reproductive mixing can also result in isolated adjacent populations in the absence of geophysical barriers (Chapman et al., 2015) in addition to environmental features restricting movement (Dudgeon et al., 2012).

### Topology of haplotype networks

Life history traits which influence the geographic structuring of evolutionary lineages could also affect the topology of a network. A star topology typically has a single widely-distributed haplotype that is positioned at the centre of the network (Jenkins, Castilho & Stevens, 2018). This central haplotype is thought to be the ancestral haplotype with the additional haplotypes linked to it differing by a single or few mutational steps (Jenkins, Castilho & Stevens, 2018). This was the predominant topology in which sharks occupy pelagic/semi pelagic habitat (eight) of which five were found in both coastal and oceanic waters. Seven reef-associated and/or demersal species also exhibited star topologies. However, four pelagic, one demersal and one reef-associated shark species with star topology did not exhibit genetic structuring. In rays, this topology was observed in three pelagic and one benthopelagic species found in both coastal and oceanic waters and all three pelagic species lacked genetic structuring. The benthopelagic species, *A. narinari*, exhibited weak but significant structuring possibly as a result of site affinity (Flowers et al., 2016). Star-like networks can indicate high connectivity, recent coalescence to a common ancestor, or population expansion. In the present study, star networks predominate among highly mobile species that lacked structuring.

In strong contrast to sharks (predominantly star topology), the majority of rays showed complex mutational topology, where several mutations separate the central and peripheral haplotypes. Eight ray species exhibiting this topology were either demersal and/or were found around coral reefs in coastal waters, including four with genetic structure. This topology was also observed in one oceanic-pelagic and one benthopelagic species, both found in coastal and oceanic habitats and both lacked structuring. The benthopelagic species, *A. ocellatus*, possibly lacked structuring because studies so far have not reported site affinity in this species. In sharks, complex mutational topologies were found in four reef-associated, two semi pelagic, one pelagic and one benthopelagic species all of which had structured populations. Therefore, shark species with complex mutational topology were structured and all of them except *Rhiz. acutus* exhibited philopatry such as seasonal residency, site fidelity, or natal philopatry.

Another difference between sharks and rays was the number of species that showed simple exclusive network topology. Three coastal-demersal rays (*B. walga*, *H. leoparda*, *G. micrura*) and one shark (*Ca. dussumieri*) showed this topology and all of them showed genetic structuring. Complex star topology was also observed in some pelagic sharks (four) and ray species (one) and one reef-associated shark. In this topology, there are multiple connections and high-frequency haplotypes (Jenkins, Castilho & Stevens, 2018). Networks with a single haplotype were observed in three sharks but not in rays. The tendency towards star mtDNA networks in sharks, and complex networks in demersal rays, may indicate a fundamental difference in phylogeographic patterns. Complex networks are common in terrestrial and freshwater organisms that inhabit highly structured habitats such as rivers and streams (*e.g.*, Schönhuth et al., 2018). Complex networks are seldom observed in marine fishes but are a recurring pattern in marine invertebrates that lack a pelagic larval stage (Bowen et al., 2020).

### Shallow coalescence

Marine teleosts tend to show very shallow coalescence in haplotype networks, indicating a shared common ancestor on a timescale much shorter than the age of the species (Grant & Bowen, 1998). Furthermore, pelagic teleosts tend to have shallower coalescence than coastal fishes (Graves, 1998). The causes for this phenomenon have been debated in the literature for over 20 years (*e.g.*, Copus et al., 2022). Here we extend these conclusions to sharks and rays, which have nearly uniformly shallow coalescence in haplotype networks (Figs. S1 & S2).

What could cause shallow mtDNA coalescences in marine teleosts, sharks and rays, relative to freshwater and terrestrial organisms? Certainly, part of the answer for sharks and rays is the low mutation rate relative to other vertebrates, initially proposed by Martin, Naylor & Palumbi (1992) and confirmed with comparisons across the Isthmus of Panama (Duncan et al., 2006; Keeney & Heist, 2006; Schultz et al., 2008). A second explanation is the vast medium of the ocean with few barriers and high biological connectivity. This is a condition shared by marine teleosts and elasmobranchs and separates their environmental regime from those of freshwater and terrestrial biota.

A third explanation for shallow coalescence, postulated for marine teleosts, is derived from r/K selection theory (MacArthur & Wilson, 1967). Marine teleost fishes are almost universally identified with an extreme version of the r-selected strategy, with high fecundity and no parental care. Thousands or millions of eggs are produced, but few survive to reproduce. This would result in a small effective population size (N_e_; Wright, 1931) relative to the census size of reproducing adults. Sweepstakes reproduction, wherein a small number of females produce most of the next generation by fortuitously placing progeny in optimal conditions for survival, would further reduce N_e_ (Hedgecock & Pudovkin, 2011). The r-selected strategy, combined with sweepstakes reproduction, could explain the shallow mtDNA coalescence in marine teleosts, but not in sharks and rays. Here, sharks and rays provide a unique insight into the genetic architecture of marine organisms. They are decidedly closer to the K-selected strategy, producing fewer progenies after a long gestation. Progeny are much further along in development, mostly arriving as miniature adults that can swim at birth. We conclude that since the r-selected teleosts and the K-selected sharks and rays both have shallow coalescence, the reproductive strategy may not drive this shared trait. The alternate explanation of high connectivity should be given greater weight and could be tested with genomic kinship analyses.

### Conservation implications

A comparison of structuring at the family level shows that two of three species within Alopiidae exhibit genetic structuring within ocean basins, followed by *Hemiscyllium* (1 of 3) and Carcharhinidae (5 of 22). Overall Φ_ST_was significant for all species belonging to 3 families–Odontaspididae, Stegostomatidae and Lamnidae. This was followed by Carcharhinidae (11 of 22), Alopiidae and *Hemiscyllium* (1 of 3) indicating structuring at some level. Structuring between ocean basins was observed in two of three species within Alopiidae, one species of *Hemiscyllium* and six species of Carcharhinidae. In rays, only Myliobatidae (1 of 2) and Dasyatidae (2 of 10) showed genetic structuring within ocean basins. However, on comparing overall Φ_ST_, significant values were observed in species from all families–Dasyatidae (5 of 10), Myliobatidae (1 of 2), Mobulidae (1 of 5) and Gymnuridae (2 of 2). Two species belonging to Dasyatidae and one belonging to Myliobatidae showed structuring across ocean basins. Hence one conclusion from our review is that management units based on political boundaries may be too small to be effective, which points to the need for transboundary collaboration (see Shiffman & Hammerschlag, 2016).

Resource managers need to understand the pattern and degree of population subdivision to prevent over-exploitation and loss of genetic diversity. The lesson from comparative phylogeography of sharks is that multiple population units with unique genetic signatures exist in most species, except in some of the large oceanic migrants. The corresponding lesson for pelagic rays is that whole ocean basins may be the scale of population units. Demersal rays may require management on a much smaller scale, based on the implications of complex haplotype networks. When population partitions exist, they are usually concordant with biogeographic boundaries such as those between ocean basins. Of course, while there will be exceptions to these trends, these can provide broad directions for management as well as point to species that urgently need genetic studies.

In at least four cases, we detected genetic separations that approach or meet the criterion for evolutionary significant units (ESUs; Moritz, 1994). The smooth butterfly ray (*Gymnura micrura*) shared no haplotypes between North and South Atlantic samples (Φ_ST_ = 0.989, *p* < 0.001). The reef-associated Indian-Ocean blue spotted maskray (*Neotrygon indica*, described in 2018 by Pavan-Kumar et al., 2018) shared no haplotypes between the Western and Central Indian Ocean (Φ_ST_ = 0.357, *p* < 0.001). Likewise, the leopard whipray (*Himantura leoparda*) and longtailed butterfly ray (*Gymnura poecilura*) showed highly significant population structure (Φ_ST_ = 0.918, *p* < 0.001 and 0.989, *p* = 0.001 respectively). It is not surprising that our survey revealed evidence for undescribed species; however, species misidentification cannot be ruled out, and where possible sequences were verified by carrying out a BLAST search on NCBI to compare and confirm the species identification. In a survey of 284 globally distributed fish species (both teleost and elasmobranch), at least 35 showed genetic evidence of cryptic evolutionary diversity (Gaither et al., 2016). In these cases, additional studies are strongly mandated to investigate the likelihood of cryptic evolutionary lineages at or below the species level. Taxonomic assignments would result in higher conservation priorities.

The conservation outlook for elasmobranchs is dire. Over the last decade, fishing has moved into deeper regions of the world’s oceans (Morato et al., 2006) and several elasmobranchs found in deep waters have been exploited (Kyne & Simpfendorfer, 2010). Many elasmobranchs that are under immense pressure from fishing activities show low levels of genetic diversity while continued overfishing can result in changes in population subdivision and loss of genetic variation (Allendorf, Luikart & Aitken, 2013; Domingues, Hilsdorf & Gadig, 2018). Globally, 1,199 species of sharks and rays have been assessed for the IUCN Red List, including a minimum of 391 (32.6%) species assigned to three threatened categories–Critically Endangered, Endangered and Vulnerable (IUCN Red List Assessment, https://www.iucnredlist.org/, Dulvy et al., 2021). A total of 299 species (24.9%) are classified as Data Deficient and 44.1% are categorised as Least Concern indicating that a majority of them need proper assessment and conservation effort (IUCN SSC Shark Specialist Group, 2019). In addition, most of these species are classified based on abundance and geographic range size, which may not necessarily be important determinants of extinction risk (Payne et al., 2011; Harnik, Simpson & Payne, 2012). In the cases considered here, large range sizes and geographic scope of populations provide some buffer from depletion and extirpation. Abundance, on the other hand, is a more serious concern for sharks and rays, especially if demographic trends lead to the erosion of genetic diversity, the necessary building blocks to adapt to a changing world.

Genetic diversity has largely been overlooked in conservation policy and fisheries management plans (Domingues, Hilsdorf & Gadig, 2018). Only about 10% of the 2014 IUCN listed shark and ray species have been studied for genetic diversity and structuring (Domingues, Hilsdorf & Gadig, 2018). Commonly caught by-catch species like pelagic sting ray (*P. violacea*) and the Critically Endangered daggernose shark (*Isogomphodon oxyrhynchus)* with narrow distribution have not yet been evaluated for discrete populations (Domingues, Hilsdorf & Gadig, 2018). It is therefore important to understand the nature of population subdivision and the type of structuring especially in those that are commercially exploited with narrow distributional ranges. This knowledge can aid in establishing policies and improving conservation plans that prevent overexploitation and aim to preserve natural genetic diversity.

## Conclusions

Our metadata analysis provides insights into how populations of sharks and rays are structured globally. It was evident that populations of sharks and rays primarily show genetic structuring across biogeographic provinces and ocean basins and, like marine teleosts, exhibit shallow coalescence in haplotype networks. No clear pattern of population subdivision could be observed for species occupying similar habitats because the reasons for structuring are complex and multifaceted. Apart from biogeographic barriers, philopatry also plays an important role in population connectivity and structure. This study was able to identify certain trends in structuring with populations of reef-associated shark species showing a higher proportion of genetic structuring across ocean basins when compared to demersal species. For rays, although non-significant, the results suggested that within basin genetic structuring could be higher for demersal species when compared to pelagic species. Network topologies of sharks were predominantly star-shaped while for rays (mostly demersal) they were complex mutational, indicating that the latter has more structured populations. Therefore, special recognition needs to be given to demersal rays which require management at a smaller scale. Since most of the shark and ray species in this study are migratory and showed genetic subdivisions among population samples, it is important that these ‘population units’ are assessed and managed individually. Conservation efforts need to move beyond political boundaries and require transboundary collaborations spanning neighbouring countries for the effective management of elasmobranchs.

## Limitations of the study

The present study has used COI sequences given their availability for a large number of sharks and rays. Other mtDNA markers like the control region could reveal a different or more nuanced view of the observed population structure patterns. The present study also did not include skates (order Rajiformes) which are an important group of egg-laying elasmobranchs. Skates could potentially show tier 1 or 2 genetic structuring at regional and local levels (Misawa et al., 2019; however, further study is needed to compare sharks, rays and skates, the three most speciose groups of elasmobranchs.

##  Supplemental Information

10.7717/peerj.15396/supp-1 Supplemental Information 1 Supplemental Figures

10.7717/peerj.15396/supp-2 Supplemental Information 2 Supplemental Table

## Additional Information and Declarations

### Data Availability

The following information was supplied regarding data availability: The cytochrome c oxidase subunit I (COI) sequences of shark species are available at NCBI GenBank (Tables S1 and S2).
