# Supplementary material for: Phylogeography of sharks and rays: a global review based on life history traits and biogeographic partitions
Source: PeerJ. 2023 Jun 1;11:e15396. doi: 10.7717/peerj.15396 (PMC10239618; doi:10.7717/peerj.15396)
Supplement: Supplemental Information 1 [file peerj-11-15396-s001.pdf]

Supplemental figures for:

Phylogeography of sharks and rays: A global review based on life history traits and biogeographic partitions

Sudha Kottillil, Chetan Rao, Brian W. Bowen, Kartik Shanker

Figures:

Figure S1 Page 2
Figure S2 Page 7

Figure S1: Median joining haplotype networks constructed using cytochrome c oxidase subunit I sequences of 40 shark species belonging to 17 genera.

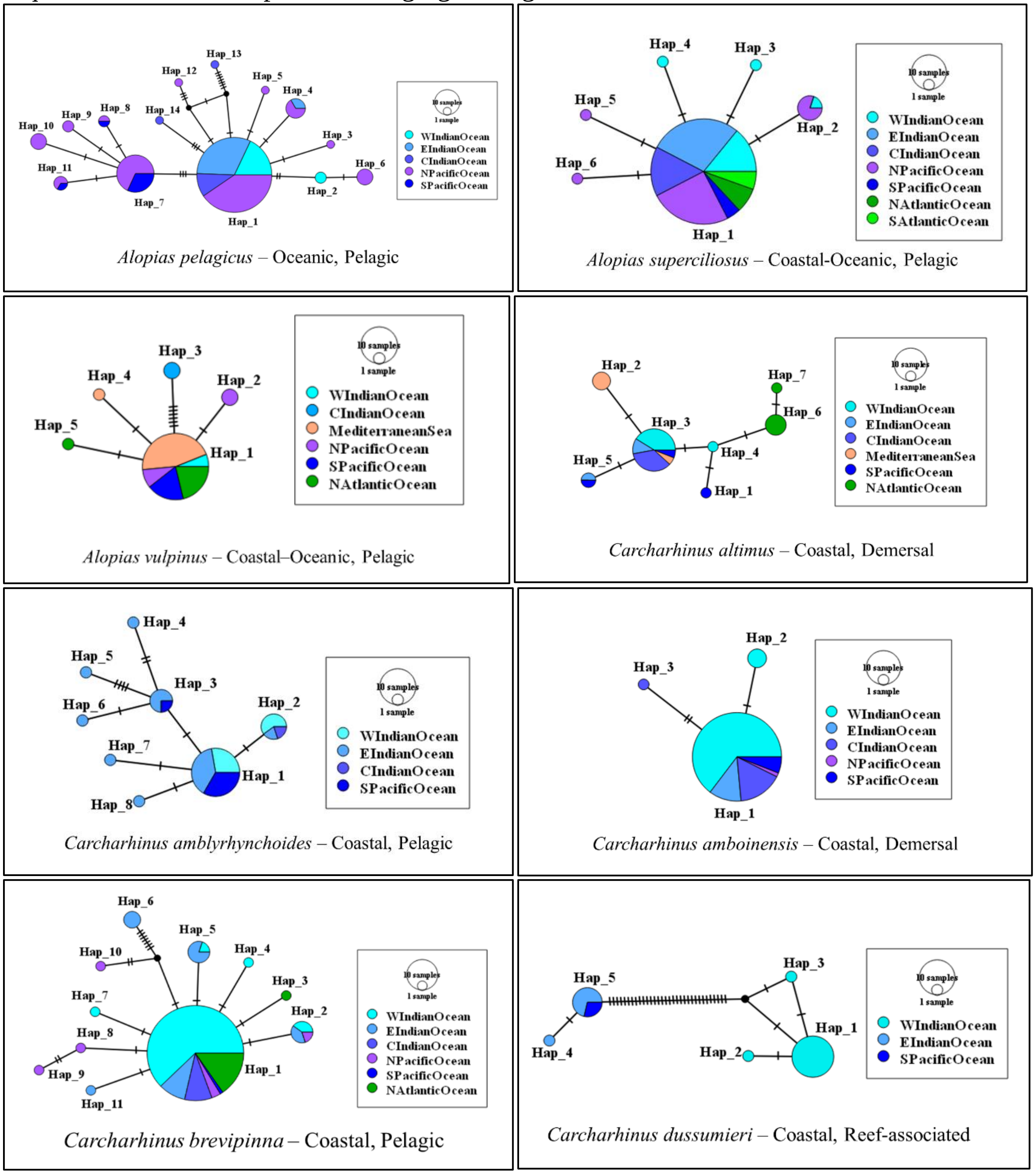

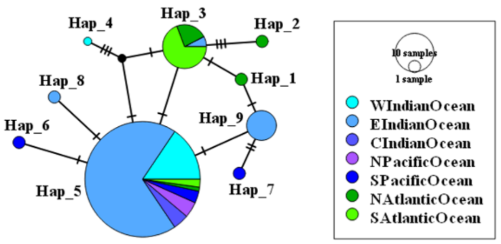

Carcharhinus falciformis – Coastal-Oceanic, Pelagic

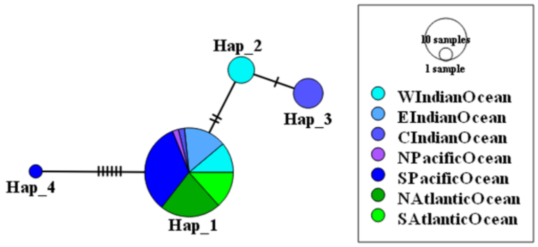

Carcharhinus leucas – Coastal, Freshwater, Reef-associated

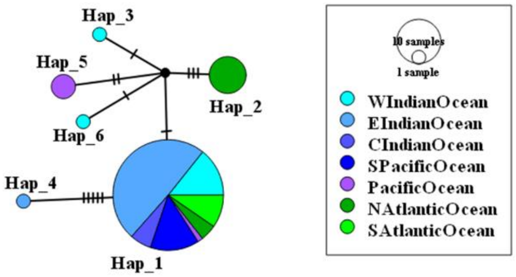

Carcharhinus limbatus – Coastal, Reef-associated

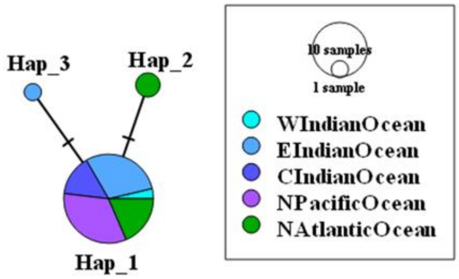

Carcharhinus longimanus – Oceanic, Pelagic

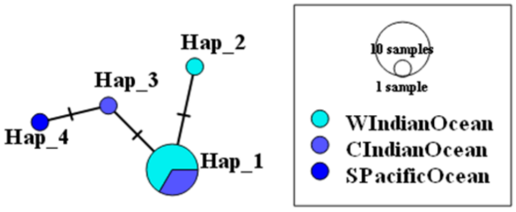

Carcharhinus macloti – Coastal, Reef-associated, Demersal

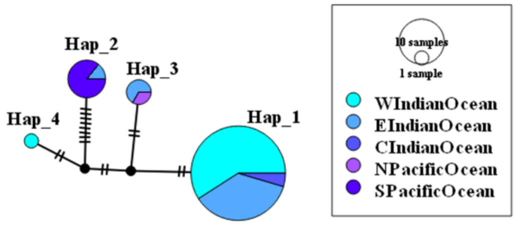

Carcharhinus melanopterus – Coastal, Reef-associated

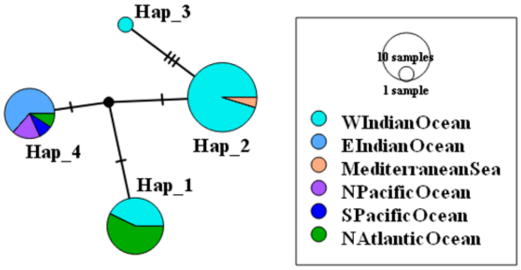

Carcharhinus plumbeus – Coastal, Benthopelagic

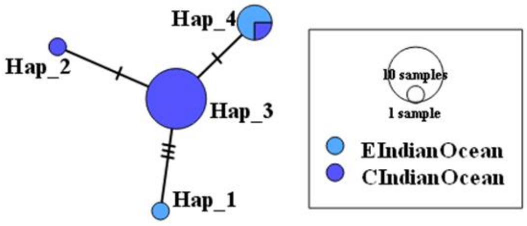

Carcharhinus sealei – Coastal, Reef-associated

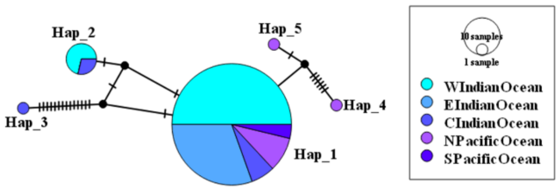

Carcharhinus sorrah – Coastal, Reef-associated

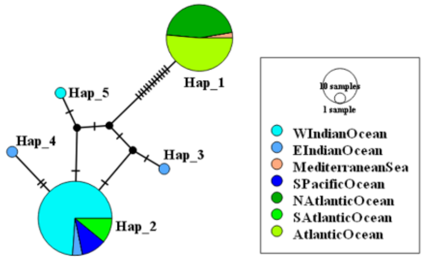

Carcharias taurus – Coastal, Reef-associated, Demersal

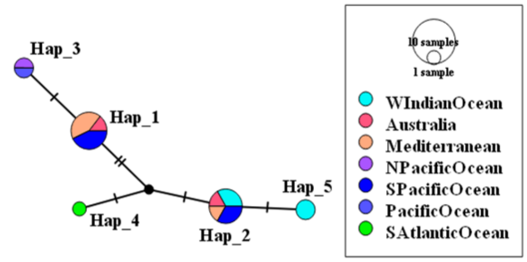

Carcharodon carcharias – Oceanic, Pelagic

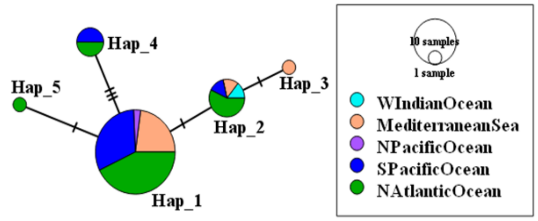

Cetorhinus maximus – Coastal-Oceanic, Pelagic

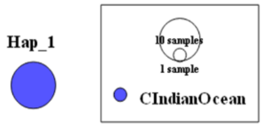

Chiloscyllium griseum – Coastal, Reef-associated, Demersal

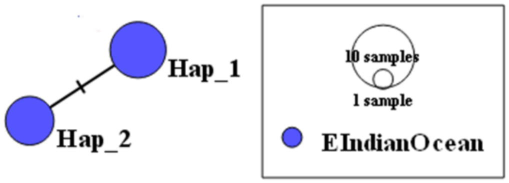

Chiloscyllium indicum – Coastal, Demersal

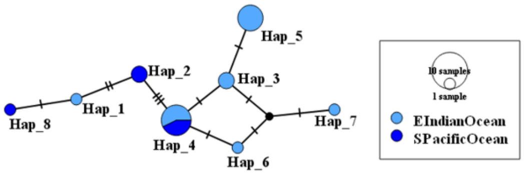

Chiloscyllium punctatum – Coastal, Reef-associated, Demersal

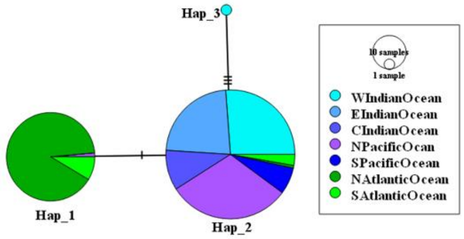

Galeocerdo cuvier – Coastal-Oceanic, Semi pelagic

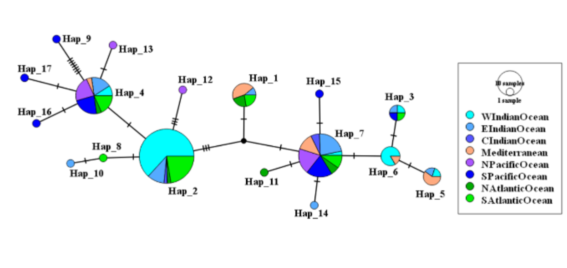

Isurus oxyrinchus – Coastal-Oceanic, Pelagic

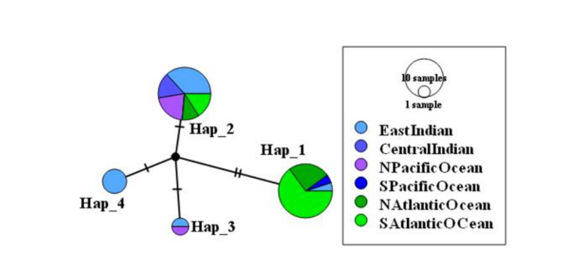

Isurus paucus – Oceanic, Pelagic

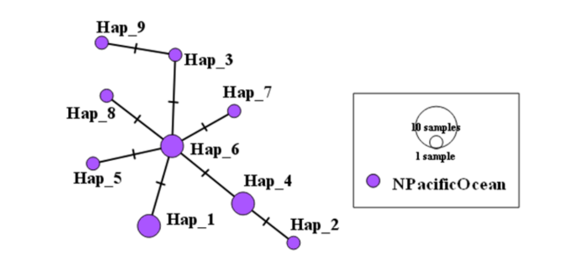

Lamna ditropis – Coastal-Oceanic, Pelagic

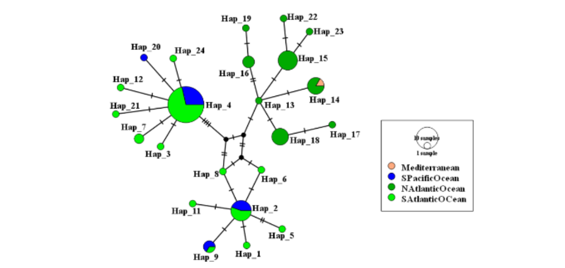

Lamna nasus – Coastal-Oceanic, Pelagic

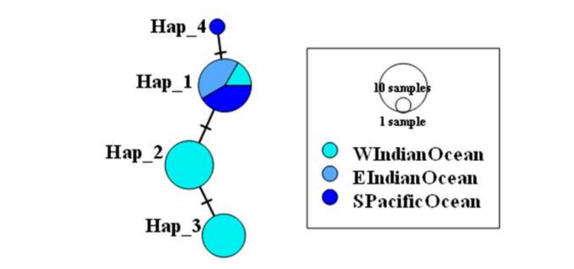

Negaprion acutidens – Coastal, Demersal

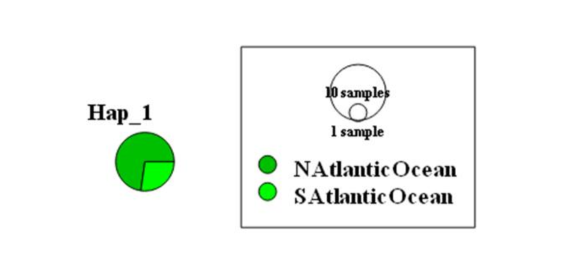

Negaprion brevirostris – Coastal, Reef-associated

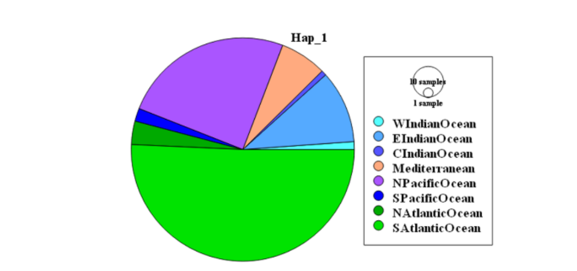

Prionace glauca – Oceanic, Pelagic

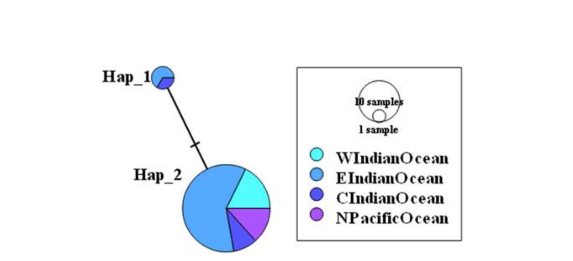

Rhincodon typus – Coastal-Oceanic, Semi Pelagic

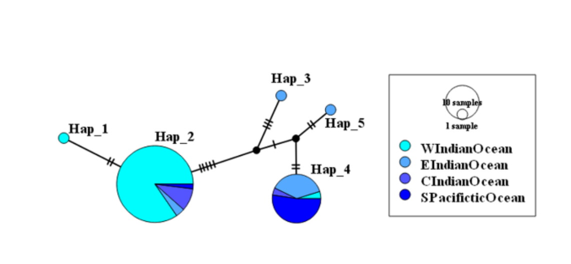

Rhizoprionodon acutus – Coastal, Benthopelagic

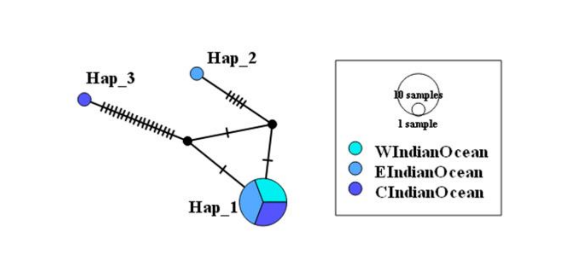

Rhizoprionodon oligolinx – Coastal, Reef-associated

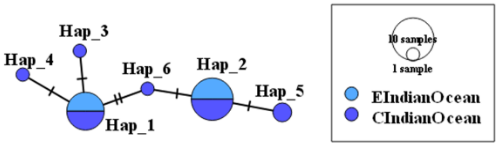

Scoliodon laticaudus

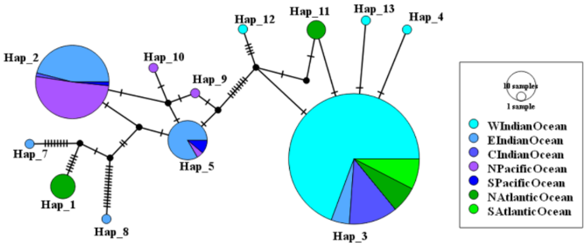

Sphyrna lewini – Coastal-Semi oceanic, Pelagic

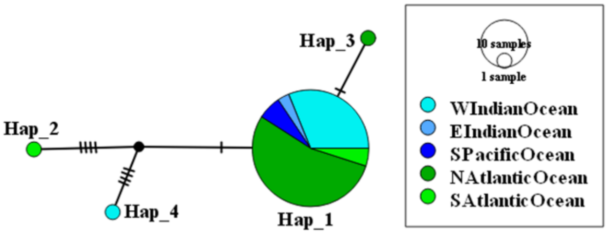

Sphyrna mokarran – Coastal-Semi oceanic, Semi pelagic

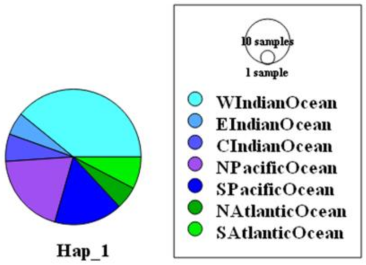

Sphyrna zygaena – Coastal-Semi oceanic, Semi pelagic

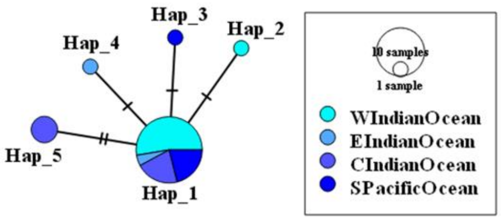

Stegosoma fasciatum – Coastal, Demersal

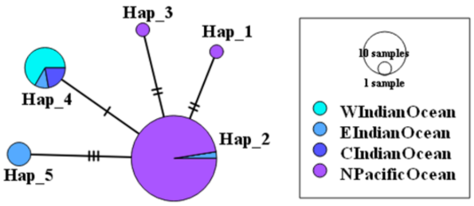

Triaenodon obesus – Coastal, Reef-associated

Figure S2: Median joining haplotype networks constructed using cytochrome c oxidase subunit I sequences of 19 ray species belonging to 11 genera.

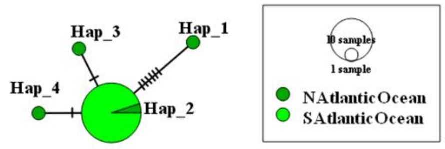

Aetobatus narinari – Coastal-Oceanic, Benthopelagic

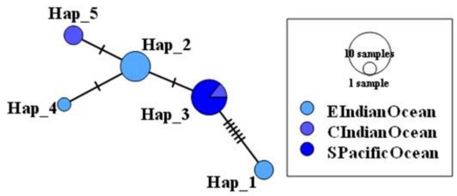

Aetobatus ocellatus – Coastal-Oceanic, Pelagic

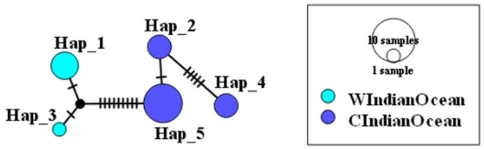

Brevitrygon imbricata – Coastal, Demersal

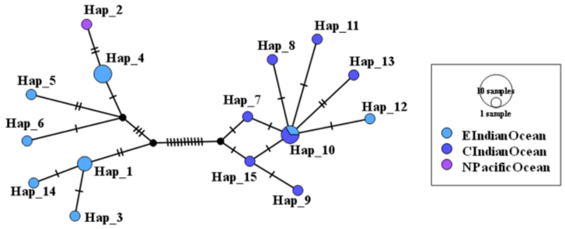

Brevitrygon walga – Coastal, Demersal

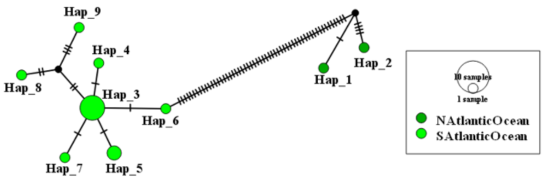

Gymnura micrura – Coastal, Demersal

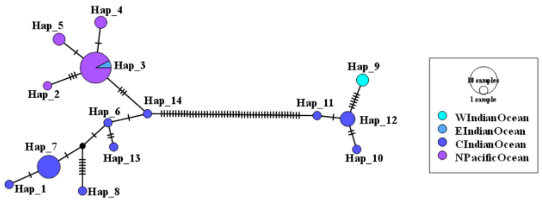

Gymnura poecilura – Coastal, Demersal

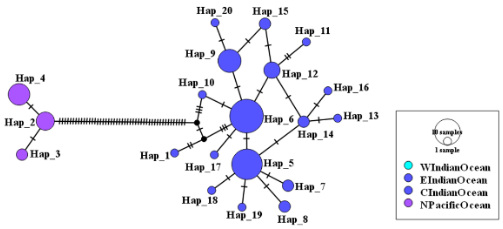

Himantura leoparda – Coastal, Demersal

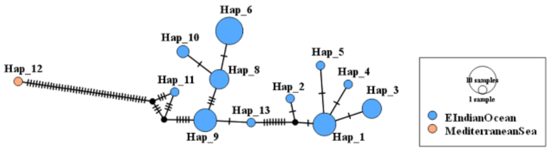

Himantura uarnak – Coastal, Demersal

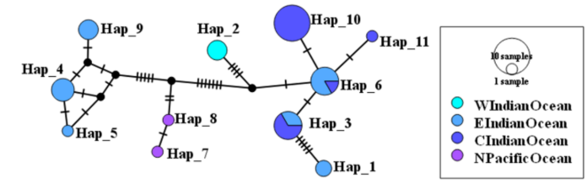

Maculabatis gerrardi – Coastal, Demersal

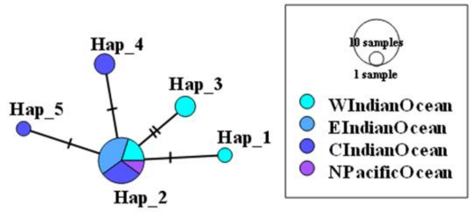

Mobular birostris – Coastal-Oceanic, Pelagic

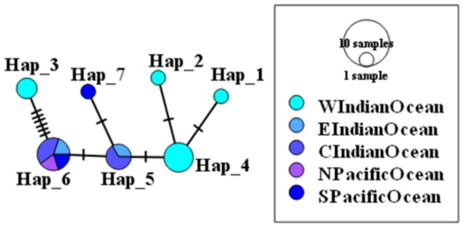

Mobular kuhlii – Coastal-Oceanic, Pelagic

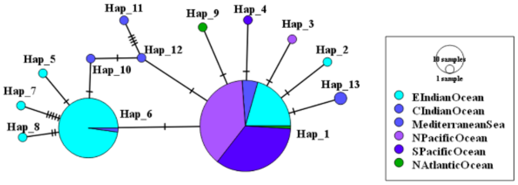

Mobula mobular – Coastal-Oceanic, Pelagic

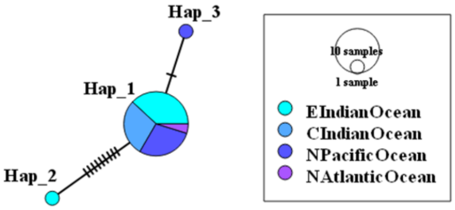

Mobular tarapacana – Coastal-Oceanic, Pelagic

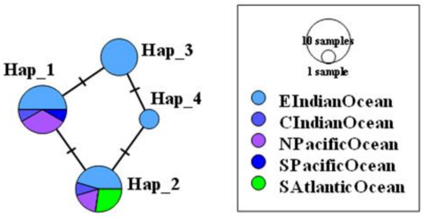

Mobular thurstoni – Coastal, Pelagic

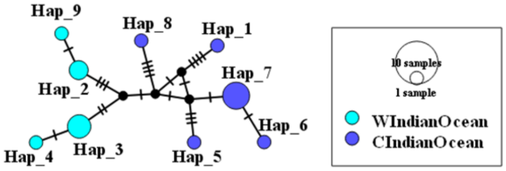

Neotrygon indica– Coastal, Reef-associated

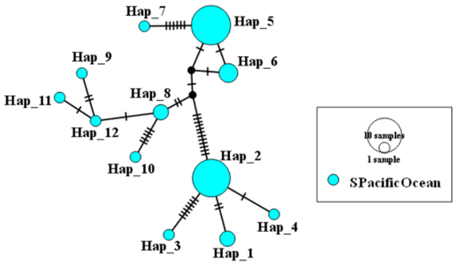

Neotrygon kulhii– Coastal, Reef-associated, Demersal

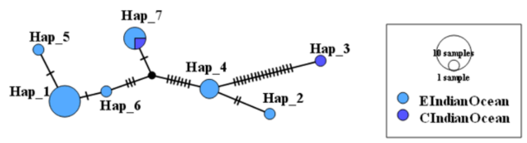

Pateobatis jenkinsii – Coastal, Demersal

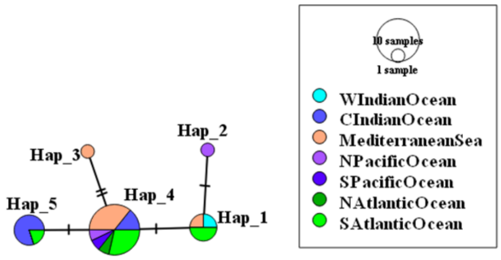

Pteroplatytrygon violacea – Oceanic, Pelagic

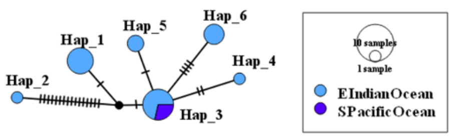

Taeniura lymma – Coastal, Reef-associated, Demersal
