# Supplementary material for: Phylogeography of sharks and rays: a global review based on life history traits and biogeographic partitions
Source: PeerJ. 2023 Jun 1;11:e15396. doi: 10.7717/peerj.15396 (PMC10239618; doi:10.7717/peerj.15396)
Supplement: Supplemental Information 2 [file peerj-11-15396-s002.pdf]

Supplemental tables for:

Phylogeography of sharks and rays: A global review based on life history traits and biogeographic partitions

Sudha Kottillil, Chetan Rao, Brian W. Bowen, Kartik Shanker

Table of Contents:

**Table S1**	Page 2
**Table S2**	Page 24
**Table S3**	Page 32
**Table S4**	Page 34

Table S1: List of authors along with the corresponding GenBank accession numbers of cytochrome c oxidase subunit I (COI) sequences of shark species (*n* = 40) that have been used in the present study. Asterix (*) indicate sequences that have not been published but is available on GenBank.

Species name (sharks)	Authors	Accession numbers of COI sequences in GenBank
Alopias pelagicus	Bineesh et al. (2016)	KF899545–KF899548 and HM239672
	Cardenosa et al. (2014)	KM218907–KM218923
	Chuang et al. (2016)	KP719248–KP719286
	Hacohen-Domene et al. (2018)*	MK188807
	Jabado et al. (2015b)	KP193429, KP193394, KP193357, KP193344, KP193340, KP193335, KP193328, KP193312, KP193299, KP193282, KP193273, KP193269, KP193264, KP193238.1, KP193237, KP193223 and KP193173
	Kumar et al. (2015)	JX978330
	Liu et al. (2013)	KF606768, KF606777, KF606787, KF606798, KF606800, KF606824, KF606838, KF606840, KF606842, KF606848, KF606850, KF606852, KF606859 and KF606941– KF606943
	Manjusha and Madhusoodanakurup (2010)*	HQ589266 and HQ589267
	Marín et al. (2018)	MH194433, MH194456 and MH194476
	Sembiring et al. (2015)	KF590224, KF590277, KF590278, KF590308–KF590313, KF590316, KF590328, KF590329, KF590332–KF590335, KF590351–KF590355, KF590439, KF590446, KF590503 and KC840948
	Spaet and Berumen (2015)	KM396948
	Velez-Zuazo et al. (2015)	KJ146022–KJ146026
	Wong et al. (2009)	FJ518964–FJ518975, FJ519183–FJ519185

Alopias superciliosus	Bineesh et al. (2016)	HM239673 and KF899549–KF899556
	Chuang et al. (2016)	KP719264–KP719280, KP719469, KP719470 and KP719487–KP719490
	Deepak et al. (2013)*	KF700944 and KF700945
	Hastings and Burton (2008)*	EU400162
	Hastings and Burton (2010)*	GU440213 and GU440214
	Jabado et al. (2015b)	KP193168, KP193210, KP193226, KP193272, KP193297, KP193301, KP193322, KP193342, KP193381, KP193400, KP193414, KP193419, KP193425, KP193436 and KP193454
	Kumar et al. (2015)	JX978336
	Liu et al. (2013)	KF606813
	Manjusha and Madhusoodanakurup (2010)*	HM990646–HM990649 and HM990651
	Sembiring et al. (2015)	KF590221, KF590226, KF590228, KF590248, KF590331, KF590337, KF590404, KF590406–KF590409, KF590417–KF590419, KF590483, KF590487–KF590490, KF590499, KF793744, KF793770 and KC840955
	da Silva Ferrette et al. (2019)	MH911331–MH911335
	Vella et al. (2017)	MF405097
	Ward et al. (2005)	DQ108329, DQ108330
	Ward et al. (2008)	EU398519–EU398521
	Wong et al. (2009)	FJ518976–FJ518986, FJ519032–FJ519034, FJ519601 and FJ519603
	Zacharia et al. (2016)*	KX063632

Alopias vulpinus	**Aguilar et al. (2020)***	**MT456029 and MT456102**
	Bineesh et al. (2016)	KF899557 and KF899558
	Cariani et al. (2017)	KT307158–KT307160
	Keskin, (2010)	HQ167643
	Lakra et al. (2011)	FJ347901–FJ347904
	Steinke et al. (2015)	JF492808 and JF492809
	Vella et al. (2017)	KY909334–KY909344
	Ward et al. (2008)	EU398522
	Wong et al. (2009)	FJ518987–FJ518998, FJ519186–FJ519190 and FJ519605
Carcharhinus altimus	Appleyard et al. (2018)	MF508661 and MF508662
	Berumen et al. (2019)	MH331689
	Bineesh et al. (2016)	KF899783–KF899788
	International Barcode of Life (2011)*	JN313266
	Jabado et al. (2015b)	KP193252, KP193277, KP193296, KP193376, KP193408, KP193441 and KP193445
	Moftah et al. (2011)	JN641206 and JN641207
	Spaet and Berumen (2015)	KM396952
	Ward et al. (2008)	EU398587–EU398589
	Wong et al. (2009)	FJ519047 and FJ519049–FJ519052
	Yokes (2016)	KY176421
Carcharhinus amblyrhynchoides	Appleyard et al. (2018)	MF508663 and MF508664
	Haque et al. (2019)	MH841990
	Jabado et al. (2015a)	KM973137–KM973141
	Moore et al. (2011)	JN034896–JN034898 and JN082181–JN082185
	Ovenden et al. (2010)	GQ227287

	Wainwright et al. (2018)	MH243097–MH243100, MH243152, MH243153, MH243161, MH243164, MH243183 and MH243233
	Ward and Holmes, (2007)	EF609307
	Ward et al. (2008)	EU398590–EU398593
Carcharhinus amboinensis	Ahmed et al. (2021)	MH230957
	Appleyard et al. (2018)	MF508671 and MF508672
	Bineesh et al. (2016)	KF899796
	Chuang et al. (2016)	KP719472
	Doukakis et al. (2011)	HQ171612 and HQ171613
	Haque et al. (2019)	MH841980, MH841984, MH841992, MH841997, MH842005, MH842006 and MH842008
	Jabado et al. (2015a)	KM973111–KM973115
	Jabado et al. (2015b)	KP193152, KP193155, KP193158, KP193162, KP193166, KP193169, KP193172, KP193177, KP193179, KP193192, KP193194, KP193218, KP193222, KP193224, KP193230, KP193244, KP193246, KP193247, KP193249, KP193250, KP193253, KP193321, KP193348, KP193380, KP193383, KP193392, KP193407, KP193422, KP193422 KP193431, KP193432, KP193434 and KP193442
	Khalil et al. (2015)*	KU366634
	Noorul-Azliana and Wahidah, (2017)*	MG644325
	Sembiring et al. (2015)	KF590340
	Spaet and Berumen (2015)	KM396937
	Steinke et al. (2015)	JF493045–JF493048
	Wainwright et al. (2018)	MH243193
	Ward et al. (2008)	EU398599, EU398600
	Wong et al. (2009)	FJ519053–FJ519056
	Zacharia et al. (2016)*	KX063634

	Zemlak et al. (2009)	DQ885075, DQ885076 and DQ884978
Carcharhinus brevipinna	Aguilar et al. (2020)*	MT456076, MT455809, MT455794, MT455455, MT455450, MT455293, MT455246, MT455095 and MT455080
	Appleyard et al. (2018)	MF508673
	Bineesh et al. (2016)	KF899797–KF899801
	Chuang et al. (2016)	KP719473–KP719475
	Deeds et al. (2014)	KF461149
	Doukakis et al. (2011)	HQ171614–HQ171641
	Haque et al. (2019)	MH841978
	Jabado et al. (2015b)	KP193451, KP193433, KP193424, KP193378, KP193374, KP193368, KP193359, KP193354, KP193346, KP193338, KP193336, KP193332, KP193327, KP193324, KP193313, KP193311, KP193307, KP193294, KP193283, KP193270, KP193267, KP193241, KP193235, KP193216, KP193198, KP193196, KP193174, KP193171, KP193144 and KM973097–KM973101
	Jamaludin and Mohd-Arshaad (2017)*	MG594062, MG594055 and MG594049
	Kumar et al. (2015)	KC175450
	Liu et al. (2013)	KF606833
	Moore et al. (2012)	JN989309
	Noorul-Azliana and Wahidah (2017)*	MG644347, MG644275–MG644277
	Semberring et al. (2017)	KF793760, KF793745, KF590405, KF590390, KF590386, KF590342, KF590293, KF590282 and KC840954
	Spaet and Berumen (2015)	KM396945
	Steinke et al. (2015)	JF493053–JF493059 and GU804990
	Wainwright et al. (2018)	MH243095, MH243105, MH243150, MH243168, MH243174, MH243176, and MH243178

	Ward et al. (2008)	EU398603–EU398601
	Wong et al. (2009)	FJ519606 and FJ519062-FJ519070
	Zacharia et al. (2016)*	KX063635
Carcharhinus dussumieri	International Barcode of Life (2010) *	GU673375, GU673386, GU673579 and GU673585
	Jabado et al. (2015a)	KM973102–KM973106
	Ward et al. (2005)	DQ108301
	Ward et al. (2008)	EU398608–EU398610
	Wong et al. (2009)	FJ519072–FJ519078
Carcharhinus falciformis	Almanza et al. (2014)	KM987408–KM987411
	Appleyard et al. (2018)	MF508674–MF508677
	Bineesh et al. (2016)	KF899803–KF899807
	Camacho and Moreno (2018)	MG837900–MG837909
	Jabado et al. (2015a)	KM973147–KM973148
	Jabado et al. (2015b)	KP193153, KP193184, KP193208, KP193233, KP193304, KP193314, KP193326, KP193375, KP193386, KP193449 and KP193452
	Johri et al. (2019)	MK092088
	Sembiring et al. (2015)	KF590281, KF590283, KF590292, KF590294-KF590301, KF590303, KF590304, KF590324–KF590326, KF590344, KF590345, KF590362–KF590373, KF590392, KF590411, KF590423–KF590426, KF590428, KF590429, KF590456, KF590484–KF590486, KF590491–KF590494, KF590497, KF590498, KF590505, KF590511, KF590515, KF793743, KF793756, KF793759, KC840952 and KC840953
	da Silva Ferrette et al. (2019)	MH719952, MH719954–MH719956, MH719958, MH719960, MH719965, MH719977 and MH719978
	Wainwright et al. (2018)	MH243106, MH243156-MH243159, MH243170, MH243172, MH243177, MH243180–MH243182 and MH243225

	Ward et al. (2008)	EU398611–EU398614
	Wong et al. (2009)	FJ519079–FJ519086, FJ519088
Carcharhinus leucas	Bineesh et al. (2016)	KF899803–KF899807
	Camacho and Moreno (2018)	MG837900–MG837909
	Jabado et al. (2015a)	KM973147–KM973148
	Jabado et al. (2015b)	KP193153, KP193184, KP193208, KP193233, KP193304, KP193314, KP193326, KP193375, KP193386, KP193449 and KP193452
	Johri et al. (2019)	MK092088
	Sembiring et al. (2015)	KF590281, KF590283, KF590292, KF590294-KF590301, KF590303, KF590304, KF590324–KF590326, KF590344, KF590345, KF590362-KF590373, KF590392, KF590411, KF590423–KF590426, KF590428, KF590429, KF590456, KF590484–KF590486, KF590491–KF590494, KF590497, KF590498, KF590505, KF590511, KF590515, KF793743, KF793756, KF793759, KC840952, KC840953 and JF493060–JF493063
	da Silva Ferrette et al. (2019)	MH719952, MH719954–MH719956, MH719958, MH719960, MH719965, MH719977 and MH719978
	Wainwright et al. (2018)	MH243106, MH243156–MH243159, MH243170, MH243172, MH243177, MH243180–MH243182, MH243184, MH243185, MH243187 and MH243225
	Ward et al. (2008)	EU398616, EU398617, EU398619, EU398618 and EU398611–EU398614
	Wong et al. (2009)	FJ518999, FJ519000–FJ519009, FJ519079-FJ519086, FJ519088 and FJ519106–FJ519109
	Wynen et al. (2009)	EU818710
Carcharhinus limbatus	Aguilar et al. (2020)*	MT455925 and MT456066
	Appleyard et al. (2018)	MF508680
	Bineesh et al. (2016)	KF899813–KF899816
	Doukakis et al. (2011)	HQ171642
	Feitosa et al. (2018)	MF686570

	Handy et al. (2013)	KF461152
	International Barcode of Life (2011) *	JN313301
	Jabado et al. (2015a)	KM973118–KM973122
	Jabado et al. (2015b)	KP193156, KP193190, KP193193, KP193195, KP193274 and KP193401
	Lakra et al. (2011)	FJ237542 and FJ237543
	Moore et al. (2011)	JN082186–JN082188
	Moore et al. (2012)	JN989310
	Noorul-Azliana and Wahidah (2017)*	MG644311 and MG644365
	Ovenden et al. (2010)	GQ227280–GQ227282
	Persis et al. (2009)	EU541307
	Ribeiro et al. (2012)	JQ365259–JQ365263
	Sembiring et al. (2015)	KF590251, KF590257, KF590259–KF590263, KF590291, KF590382, KF590500, KF590506, KF793730–KF793732, KF793747 and KF793748
	da Silva Ferrette et al. (2019)	MH911050 and MH911051
	Spaet and Berumen (2015)	KM396943
	Steinke et al. (2015)	JF493064 and JF493065
	Ward et al. (2008)	EU398620–EU398625
	Wong et al. (2009)	FJ519110–FJ519116 and FJ519613–FJ519616
	Zemlak et al. (2009)	DQ884979–DQ884981
Carcharhinus longimanus	Bineesh et al. (2016)	KF913239 and KF913240–KF913242
	Hastings and Burton (2010)*	GU440259
	Jabado et al. (2015a)	KM973123–KM973126
	Jabado et al. (2015b)	KP193281
	Moore et al. (2012)	JN989311
	Sembiring et al. (2015)	KF590242, KF590246, KF590412, KF590413, KF590427, KF590501, KF590504 and KF793726
	Ward et al. (2007)	EF609312

	Ward et al. (2008)	EU398627–EU398629
	Wong et al. (2009)	FJ518919–FJ518928, FJ519117–FJ519119, FJ519368 and FJ519617–FJ519620
Carcharhinus macloti	Bineesh et al. (2016)	KF913239–KF913242
	Jabado et al. (2015)	KM973123–KM973126
	Moore et al. (2012)	JN989311
	Ward et al. (2007)	EF609312
	Ward et al. (2008)	EU398628 and EU398629
Carcharhinus melanopterus	Appleyard et al. (2018)	MF508681
	Bineesh et al. (2016)	KF899824, KF899823 and MK422108
	Hubert et al. (2012)	JQ431553
	International Barcode of Life (2010)*	HM387141
	International Barcode of Life (2011)*	JN313260 and JN313261
	Jabado et al. (2015a)	KM973092–KM973096
	Jabado et al. (2015b)	KP193207, KP193215, KP193231, KP193236, KP193260, KP193341, KP193362, KP193371, KP193410, KP193413, KP193418, KP193421, KP193443, KP193453 and KP193157
	Moore et al. (2011)	JN082189 and JN082190
	Segura-Garcia and Yain Tun (2018)*	MH235615
	Sembiring et al. (2015)	KF590379, KF590380, KF590383, KF590389, KF793769 and KF793772
	Spaet and Berumen (2015)	KM396936
	Wainwright et al. (2018)	MH243165 and MH243191
	Ward et al. (2007)	EF609313
	Ward et al. (2008)	EU398630–EU398633
	Wong et al. (2009)	FJ519120–FJ519127

Carcharhinus plumbeus	Aguilar et al. (2020)*	MT455294, MT455848, MT455995, MT456051 and MT456060
	Appleyard et al. (2018)	MF508682
	Chuang et al. (2016)	KP719303 sand KP719304
	Doukakis et al. (2011)	HQ171649–HQ171652
	Jabado et al. (2015a)	KM973127–KM973131
	Jabado et al. (2015b)	KP193149, KP193151, KP193178, KP193189, KP193191, KP193206, KP193212, KP193229, KP193279, KP193286, KP193317, KP193365, KP193369, KP193403 and KP193409
	Sembiring et al. (2015)	KF590222, KF590225 and KF590227
	Spaet and Berumen (2015)	KM396951
	Steinke et al. (2015)	JF493067–JF493070
	Ward et al. (2008)	EU398638 and EU398639
	Wong et al. (2009)	FJ519152–FJ519156 and FJ519623
Carcharhinus sealei	Bineesh et al. (2016)	MG644363, MK422103 and MK422104–MK422106
	Sembiring et al. (2015)	KF590375–KF590378, KF590393. KF590395 and KF793767
	Wainwright et al. (2018)	MH243171
	Ward et al. (2008)	EU398640–EU398644
Carcharhinus sorrah	Appleyard et al. (2018)	MF508683
	Ahmed et al. (2021)	MH429287 and MH429296
	Bineesh et al. (2016)	KF899817–KF899822
	Chuang et al. (2016)	KP719305, KP719603KP719611 and KP719830
	Doukakis et al. (2011)	HQ171653–HQ171667
	Haque et al. (2019)	MH841979, MH841999 and MH842000
	International Barcode of Life (2010)*	JN313264
	Jabado et al. (2015a)	KM973132–KM973136

	Jabado et al. (2015b)	KP193145, KP193147, KP193161, KP193163, KP193164, KP19317, KP193180–KP193182, KP193221, KP193225, KP193232, KP193234, KP193239, KP193245, KP193248, KP193254, KP193259, KP193265, KP193268, KP193271, KP193275, KP193284, KP193288, KP193290, KP193295, KP193308, KP193315, KP193325, KP193351, KP193364, KP193366, KP193372, KP193388, KP193391, KP193398, KP193412, KP193423, KP193435 and KP193448
	Khalil et al. (2015)*	KU366614, KU366624, KU366628 and KU366630
	Kumar et al. (2013)	JX978335
	Moore et al. (2011)	JN082191 and JN034904
	Sembiring et al. (2015)	KF590258, KF590268, KF590269, KF590343, KF590356, KF590357, KF590384, KF590440, KF590442–KF590444, KF590458, KF793728, KF793746, KF793761, KF793763, KF793764 and KC840949–KC840951
	Spaet and Berumen (2015)	KM396941
	Wainwright et al. (2018)	MH243104, MH243173, MH243129, MH243198, MH243206, MH243208, MH243212 and MH243221
	Ward et al. (2008)	EU398645
	Wong et al. (2009)	FJ519167–FJ519172
	Zacharia et al. (2016)*	KX063636
Carcharodon carcharias	Hastings and Burton (2010)*	GU440260
	Iglesia et al. (2014)	KM212005
	International Barcode of Life (2011)*	JN312956
	Keskin (2010)*	HQ167639
	Steinke et al. (2015)	JF493076
	Vella et al. (2017)	KY909355
	Ward et al. (2005)	DQ108328
	Ward et al. (2008)	FJ518939–FJ518944 and EU398646
	Yokes (2016)*	KY176701

	Zemlak et al. (2009)	DQ884985–DQ884987
Carcharias taurus	Aguilar et al. (2020)*	MT455256 and MT456108
	Jabado et al. (2015a)	KM973199
	Keskin (2010)*	HQ167637
	Momigliano et al. (2015)	KR003980–KR003983
	Steinke et al. (2015)	JF493071–JF493075
	Wong et al. (2009)	FJ519624 and FJ519628–FJ519699
Cetorhinus maximus	Cariani et al. (2017)	KT307166–KT307170
	Keskin (2010)*	HQ167642
	Wong et al. (2009)	FJ519292–FJ519322, FJ519325–FJ519328 and FJ519335–FJ519339
	Yokes (2016)*	KY176425 and KY176702
Chiloscyllium griseum	Bamaniya et al. (2016)*	KJ093271–KJ093276
	Bineesh et al. (2016)	KF899625–KF899628
	Kumar et al. (2015)	KC175451
	Steinke et al. (2009)	FJ583140 and FJ583141
Chiloscyllium indicum	Ward et al. (2008)	EF609325 and EU398680–EU398692
Chiloscyllium punctatum	International Barcode of Life (2011)*	HQ955999, HQ956000 and JN313263
	Segura-Garcia and Yain Tun (2018)*	MH235621
	Steinke et al. (2009)	FJ583142–FJ583144
	Ward et al. (2007)	EF609326
	Ward et al. (2008)	EU398697–EU398707
Galeocerdo cuvier	Aguilar et al. (2020)*	MT455235, MT455562 and MT455748
	Ahmed et al. (2021)	MN013428 and MH429290
	Appleyard et al. (2018)	MF508685
	Bineesh et al. (2016)	KF899429–KF899436, HM239674
	Chuang et al. (2016)	KP719330, KP719613 and KP719614

	da Silva Ferrette et al. (2019)	MH911009–MH911011
	Deepak et al. (2011)*	JN657190
	Doukakis et al. (2011)	HQ171668–HQ171676
	Feitosa et al. (2018)	MF686571 and MF686584
	Haque et al. (2019)	MH841986
	Jabado et al. (2015a)	KM973198
	Jabado et al. (2015b)	KP193148, KP193160, KP193187, KP193200, KP193205, KP193214, KP193261, KP193262, KP193289, KP193309, KP193329, KP193349, KP193367, KP193370 and KP193385
	Liu et al. (2013)	KF606776 and KF606778
	Pirog (2019)	MK359168–MK359171
	Sarmiento-Camacho et al. (2018)	MG837930–MG837933
	Sembiring et al. (2015)	KF590290 and KF590299
	Spaet and Berumen (2015)	KM396946
	Vysakh (2019)*	MN017113
	Wainwright et al. (2018)	MH243175, MH243201, MH243219 and MH243228
	Ward et al. (2008)	EU398785–EU398788
	Wong et al. (2009)	FJ519801–FJ519959
	Zacharia et al. (2014)*	KJ475202
	Zemlak et al. (2009)	DQ885011–DQ885013, DQ885091 and DQ885091
Isurus oxyrinchus	Aguilar et al. (2020)*	MT456007
	Barbuto et al. (2010)	FM164462, FM164463, FM164465, FM164467, FM164468, FM164470, FM164473, FM164474 and FM164475
	Bengil et al. (2019)	KY290584 and MG214784
	Bineesh et al., (2016)	KF899536–KF899541
	Chuang et al. (2016)	KP719234–KP719246

	da Silva Ferrette et al. (2019)	MH911342–MH911346, MH194507, MH719959, MH719963, MH719966 and MH719979
	de Carvalho (2014)	KF771232
	Jabado et al. (2015b)	KP193143, KP193150, KP193176, KP193183, KP193197, KP193203, KP193204, KP193209, KP193213, KP193240, KP193251. KP193255, KP193258. KP193266, KP193291, KP193303, KP193330, KP193331, KP193347, KP193352, KP193353, KP193373, KP193377, KP193379, KP193384, KP193387, KP193389, KP193390, KP193395, KP193396, KP193402, KP193427, KP193430, KP193439 and KP193440
	Keskin (2010)*	HQ167640
	Kumar et al. (2015)	JX978337
	McCusker et al. (2013)	KC015501 and KC015502
	Ribeiro et al. (2012)	JX124792 and JX034003–JX034006
	Sembiring et al. (2015)	KF590247, KF590279, KF590306, KF590307, KF590330, KF590336, KF590338, KF590339, KF590381, KF590420, KF590421, KF590422, KF590437, KF590438. KF793721 and KF793722
	Steinke et al. (2015)	JF493694–JF493697
	Townsend and McCracken (2015)	KT444008
	Velez-Zuazo et al. (2015)	KJ146030–KJ146038
	Vella et al. (2017)	KY909421–KY909429
	Ward et al. (2008)	EU398889–EU398898 and EU869822
	Wong et al. (2009)	FJ518945–FJ518952 and FJ519206–FJ519210
	Zacharia et al. (2016)*	KX063638
Isurus paucus	Bineesh et al., (2016)	KF899542–KF899544
	Chuang et al. (2016)	KP719247
	da Silva Ferrette et al. (2019)	MH911336–MH911341, MH719923–MH719928, MH719928, MH719931, MH719932, MH719939, MH719940, MH719964 and MH719976

	Sembiring et al. (2015)	KF590434–KF590436, KF590460, KF590464, KF590468, KF590472, KF590474, KF590478, KF590495 and KF590496
	Ward et al. (2008)	EU398899 and EU398900
	Wong et al. (2009)	FJ519010, FJ519012–FJ519019 and FJ519627–FJ519629
Lamna ditropis	Elz et al. (2013)	KF918878
	Mecklenburg et al. (2011)	HQ712511
	Wong et al. (2009)	FJ519020–FJ519031
	Zhang and Hanner (2011)	JF952773
Lamna nasus	Keskin (2010)*	HQ167641
	Velez-Zuazo et al. (2015)	KJ146039–KJ146041
	Wong et al. (2009)	FJ519648–FJ519724
Negaprion acutidens	Appleyard et al. (2018)	MF508686 and MF508686
	International Barcode of Life (2010)*	GU674232
	Jabado et al. (2015a)	KM973164 and KM973165
	Jabado et al. (2015b)	KP193167, KP193186, KP193199, KP193211, KP193243, KP193280, KP193293, KP193300, KP193318, KP193320, KP193323, KP193406, KP193428, KP193437, KP193428, KP193437 and KP193438
	Spaet and Berumen (2015)	KM396935
	Ward et al. (2005)	DQ108284
	Ward et al. (2008)	EU398935–EU398940
	Wong et al. (2009)	FJ519224–FJ519225
Negaprion brevirostris	Wong et al. (2009)	FJ519226–FJ519235 and FJ519631
Prionace glauca	Almerón et al. (2018)	MG703523–MG703531, MG703533, MG703534 and MG703536
	Barbuto et al. (2010)	FM164482, FM164428, FM164458 and FM164459
	Bénard-Capelle et al. (2015)	KP975821–KP975822
	Bineesh et al. (2016)	KF899650–KF899653

Cariani et al. (2017)	KT307365 and KT307366
Chuang et al. (2016)	KP719342, KP719343 and KP719345–KP719450
da Silva Ferrette et al. (2019)	MH911185–MH911253 and MH719774–MH719984
Ferrito et al. (2019)	MN447694–MN447697
Giovos et al. (2020)	MN641758, MN641761, MN641763, MN641766, MN641769, MN641770, MN641777, MN641781, MN641784, MN641785, MN641788, MN641789, MN641798, MN641799, MN641800 and MN641801
Gkafas et al. (2015)	KP192409
Hastings and Burton (2008)*	EU400175
International Barcode of Life (2011)*	JN312503–JN312505
Jabado et al. (2015b)	KP193159, KP193306, KP193339, KP193350, KP193446 and KP193455
Liu et al. (2013)	KF606771, KF606774, KF606779, KF606788, KF606807, KF606817, KF606823, KF606830, KF606846 and KF606857
Marín et al. (2018)	MH194440, MH194441, MH194480, MH194481, and MH194484
McCuster et al. (2013)	KC015829–KC015834
Nicolé et al. (2011)	GU324182
Oliveira et al. (2016)	KX586223–KX586225
Sembiring et al. (2015)	KF590231–KF590241, KF590244, KF590245, KF590280, KF590302, KF590459, KF590461 –KF590463, KF590465 - KF590467, KF590470, KF590471, KF590473, KF590475–KF590477, KF590479–KF590482, KF793717–KF793720, KF793723–KF793725, KF793727, KF793750, KF793751, KF793758 and KF793771
Velez-Zuazo et al. (2013)	KJ146042–KJ146044
Vella et al. (2017)	KY909463–KY909469
Wainwright et al. (2018)	MH243131–MH243139, MH243143, MH243144, MH243160, MH243163 and MH243166

	Ward et al. (2005)	DQ108286, DQ108288 and DQ108289
	Ward et al.,(2008)	EU869837
	Wong et al. (2009)	FJ518955–FJ518963, FJ519237–FJ519243, FJ519632 and FJ519633
	Yokes (2016)*	KY176584
Rhincodon typus	Bineesh et al. (2016)	KF899632–KF899634
	Haque et al. (2019)	MH842010
	Hastings and Burton (2010)*	GU440502
	Jabado et al. (2015a)	KM973184
	Marín et al. (2018)	MH194467
	Steinke et al. (2015)	HQ945887–HQ945889
	Toha et al. (2020)	MN759737–MN759764
	Ward et al. **(**2008)	EU398993
	Wong et al. (2009)	FJ519244–FJ519252
Rhizoprionodon acutus	Bineesh et al. (2016)	KF899684–KF899688 and MK422110
	Doukakis et al. (2011)	HQ171695–HQ171734
	Jabado et al. (2015a)	KM973176, KM973178 and KM973180
	Kumar et al. (2015)	JX978338
	Sembiring et al. (2015)	KF590219, KF590220, KF590223, KF590229, KF590256, KF590264–KF590267, KF793733–KF793737 and KF793749
	Spaet and Berumen (2015)	KM396933
	Wainwright et al. (2018)	MH243103 and MH243140
	Ward et al. (2005)	DQ108275–DQ108278 and DQ108290
	Wong et al. (2009)	FJ519253
Rhizoprinodon oligolinx	Andriyono et al. (2020)	MH085756
	Jabado et al. (2015a)	KM973185–KM973188

	Wainwright et al. (2018)	MH429294, MH429295, MH311279, MH311281, MH311285, MH243096, MH243142, MH243149, MH243151 and MH243154
Scoliodon laticaudus	Ahmed et al. (2021)	MH230956, MH429292 MH087056 and MH087057
	Bineesh et al. (2016)	KF899694–KF899700
	Habib et al. (2019)*	MN458374
	Haque et al. (2019)	MH841994
	International Barcode of Life (2011) *	HQ956149
	Kumar et al. (2015)	JX978326–JX978328 and KC175448
	Wainwright et al. (2018)	MH243114–MH243122
Sphyrna lewini	Aguilar et al. (2020)*	MT455337, MT455567 and MT456041
	Ahmed et al. (2021)	MH230949, MH429288 and MH429289
	Alghozali et al. (2019)	LC422406–LC422410
	Appleyard et al. (2018)	MF508689–MF508691
	Bineesh et al. (2016)	KF899746–KF899751, MK422114 and HM239675
	de Oliveira Ribeiro et al. (2012)	JQ365581–JQ365585
	Doukakis et al. (2011)	HQ171735–HQ171776
	Hastings and Burton (2010)*	GU440527
	Jabado et al. (2015a)	KM973194–KM973197
	Jabado et al. (2015b)	KP177233–KP177307
	Sarmiento-Camacho et al. (2018)	MG837998–MG838000
	Segura-Garcia and Yain Tun (2018)*	MH235722 and MH235723
	Sembiring et al. (2015)	KF590254, KF590255, KF590271 - KF590276, KF590305, KF590315, KF590317–KF590323, KF590327, KF590347, KF590348, KF590358, KF590359, KF590394, KF590431, KF590449–KF590455, KF793729, KF793738 - KF793742, KF793753 and KF793757

	Steinke et al. (2015)	JF494559–JF494561
	Sukumar et al. (2020)	MH593266–MH593272 and MH593283–MH593289
	Wainwright et al. (2018)	MH243101, MH243108, MH243124, MH243126, MH243128, MH243141, MH243147 and MH243148
	Ward et al. (2008)	EU399011–EU399014
	Wong et al. (2009)	FJ519373–FJ519458 and FJ519635–FJ519637
	Zemlak et al. (2012)	DQ885056, DQ885057 and DQ885126–DQ885127
	Zhang and Hanner (2012)	FJ237951–FJ237956
Sphyrna mokarran	Appleyard et al. (2018)	MF508693
	da Silva Ferrette et al. (2019)	MH911047, MH911048 and MK188802
	Feitosa et al. (2018)	MF686574
	Hacohen-Domene et al. (2018)*	MK188824
	Jabado et al. (2015a)	KM973189–KM973193
	Jabado et al. (2015b)	KP193257 and KP177308–KP177317
	Moore et al. (2012)	JN989316
	Spaet and Berumen (2015)	KM396934
	Ward et al. (2008)	EU399015–EU399017
	Wong et al. (2009)	FJ519459–FJ519488, FJ519639 and FJ519641
Sphyrna zygaena	Aguilar et al. (2020)*	MT455636 and MT455637
	Almerón-Souza et al. (2018)	MG703560
	Appleyard et al. (2018)	MF508694
	Bineesh et al. (2016)	KF899752–KF899757
	Chuang et al. (2016)	KP719454–KP719458
	International Barcode of Life (2011)*	HM909793

	Jabado et al. (2015b)	KP193202, KP193220, KP193242, KP193256, KP193263, KP193278, KP193292, KP193302, KP193305, KP193316, KP193333, KP193343, KP193355, KP193356, KP193360, KP193361, KP193397, KP193404, KP193417, KP193426, KP193444 and KP177224–KP177232
	Liu et al. (2013)	KF606765, KF606766, KF606815 and KF606858
	Marín et al. (2018)	MH194422, MH194471 - MH194475, MH194487, MH194504, MH194431 and MK070513
	Mwangi et al. (2019)*	MK545100 and MK545101
	Sembiring et al. (2015)	KF590243, KF590430, KF590432 and KF590433
	Steinke et al. (2015)	JF494562–JF494564
	Velez-Zuazo et al. (2015)	KJ146045
	Ward et al. (2008)	EU399018
	Wong et al. (2009)	FJ519525–FJ519544
Stegostoma fasciatum	Bineesh et al. (2016)	HM239676
	Doukakis et al. (2011)	HQ171777
	Dudgeon et al. (2009)	FJ178398–FJ178402
	Jabado et al. (2015a)	KM973166–KM973170
	Jabado et al. (2015b)	KP193228 and KP193405
	Sembiring et al. (2015)	KF590349
	Ward et al. (2008)	EU399050–EU399053
	Zacharia et al. (2016)*	KX063639
Triaenodon obesus	Bineesh et al. (2016)	KF899764–KF899767
	Diaz-Ferguson et al. (2016)	KT275201–KT275239
	Sembiring et al. (2015)	KF590341, KF590361 and KF590388
	Sijo et al. (2019)*	MK492925
	Spaet and Berumen (2015)	KM396947
	Wong et al. (2009)	FJ519288 and FJ519289

	Yambot et al. (2012)*	KC970512

Table S2. List of authors along with the corresponding GenBank accession numbers of cytochrome c oxidase subunit I (COI) sequences of ray species (*n* = 19) that have been used in the present study. Asterix (*) indicate sequences that have not been published but is available on GenBank.

Species name (rays)	Authors	Accession numbers of COI sequences in GenBank
Aetobatus narinari	Richards et al. (2009)	FJ812200–FJ812203
	Sales et al. (2019)	MK340509–MK340527
Aetobatus ocellatus	Berthe et al. (2016)	KT208286–KT208291
	Hartoko et al. (2020)	LC505460
	John et al. (2020)	MG774904
	Lim et al. (2015)	KM073028 and KM073029
	Mohd-Arshaad et al. (2016)*	KX219586
	Ravi et al. (2019)*	MK422135–MK422137
	Ward et al. (2008)	EU398508
Brevitrygon imbricata	Bhagyalekshmi and Kumar (2021)	MT776900
	Bhaskar and Das (2018)*	MK331965 and MK331966
	Bineesh et al. (2016)	KF899354–KF899356 and KF899512–KF899521
	Habib et al. (2017a)	MF611582
	International Barcode of Life (2010)*	GU673374
	Last et al. (2013)	KF604909 and KF604917
	Prasannakumar et al. (2008)*	FJ384709
	Rabaoui et al. (2019)	KU317892 and KU317893
Brevitrygon walga	Ahmed et al. (2021)	MH429304, MH429305, MH429306, MH429310, MN013425, MH230948 and MN083136
	Basumatary et al. (2017)	MF495712 and MF495713

	Habib et al. (2017b)*	MF614769
	International Barcode of Life (2011)*	HQ955940 and HQ955948
	Last et al. (2013)	KF604912
	Lim et al. (2015)	KM072994 and KM072995
	Ward et al. (2008)	EU398872–EU398876
Gymnura micrura	International Barcode of Life (2010)*	HQ575754 and HQ575767
	Sales et al. (2019)	MN105752, MN105825–MN105830, MN105832, MN105833 and MN105836–MN105838
Gymnura poecilura	Ahmed et al. (2021)	MH230947
	Basumatary et al. (2017)	MF495714 and MF495715
	Bineesh et al. (2016)	KF899442, KF899443, KF899444, KF899445 and KF89944
	Habib et al. (2019)*	MN458407
	Haque et al. (2019)	MH842007
	John et al. (2020)	MG774900, MG774901, MG774908, MG774909, MG774911, MG774912, MG774920, MG774921, MG792071–MG792073.1, MG792081, MG792085–MG792087, MG792089 and MG792090
	Kumar et al. (2015)	JX978320–JX978324
	Rabaoui et al. (2019)	KU499632 and KU499714
	Ward et al. (2008)	EU398804
Himantura leoparda	Arlyza et al. (2013a)	JX263361–JX263417
	Bineesh et al. (2016)	KF899353 and KF899500–KF899502
	John et al. (2020)	MG792078, MG792101, MG792112, MG792113, MG792124, MG792125, MG774902, MG774903, MG774913.1, MG774915 and MG774922
	Lim et al. (2015)	KM072996–KM072998

	Ravi et al. (2019)*	MK422130
	Steinke et al. (2015)	JF493651 and JF493652
Himantura uarnak	Arlyza et al. (2013a)	JX263337–JX263360
	Bineesh et al. (2016)	KF899507–KF899511
	Cerutti-Pereyra et al. (2012)	JQ765509, JQ765519–JQ765530, JQ765594 and JQ765595
	John et al. (2020)	MG792110 and MG792123
	Lim et al. (2015)	KM072999 and KM073000
	Yokes (2016)*	KY176708
Maculabatis gerrardi	Ahmed et al. (2021)	MH230945
	Arlyza et al. (2013a)	JX263423 and JX263424
	Bineesh et al. (2016)	KF899364, KF899476–KF899487
	Kumar et al. (2015)	KC175446 and JX978325
	Lim et al. (2015)	KM073002 and KM073003
	Ravi et al. (2019)*	MK422126–MK422129
	Segura-Garcia and Yain Tun (2018)*	MH235645
	Steinke et al. (2015)	JF493648–JF493650
	Wainwright et al. (2018)	MH243129 and MH243130
	Ward et al. (2005)	DQ108164 and DQ108177
	Ward et al. (2008)	EU398840–EU398840
	Zacharia et al. (2014)*	KJ475200
Mobula birostris	Bineesh et al. (2016)	KF899564–KF899569
	Poortvliet et al. (2015)	KM364883 and KM364884
	Steinke et al. (2011)	JF493862–JF493866

	Ward et al. (2008)	EU398902–EU398904
Mobula kuhlii	Bineesh et al. (2016)	KF899581–KF899583
	Haque et al. (2019)	MH841993
	International Barcode of Life (2010)*	GU673390
	Lim et al. (2015)	KM073011
	Poortvliet et al. (2015)	KM364893–KM364896
	Segura-Garcia and Yain Tun (2018)*	MH235671
	Steinke et al. (2011)	JF493895–JF493899
	Ward et al. (2008)	EU398907
Mobula mobular[1]	Ahmed et al. (2021)	MH230952
	Bamaniya et al. (2016)*	KJ093277 and KJ093278
	Bineesh et al. (2016)	KF899570
	Cariani et al. (2017)	KT307247
	Chuang et al. (2016)	KP719334–KP719338
	Haque et al. (2019)	MH842002
	International Barcode of Life (2010)*	GU674092, GU674234–GU674236, GU674396, GU674397, GU674399 and GU674401
	International Barcode of Life (2011)*	HQ956137
	John et al. (2020)	MG774910
	Kumar et al. (2015)	KC175452
	Manjusha and Madhusoodanakurup (2010)*	HQ589285 and HQ589286
	Marín et al. (2018)	MH194451–MH194453, MH194455, MH194457, MH194461, MH194464, MH194469 and MK070516
	Poortvliet et al. (2015)	KM364889–KM364892, KM364897 and KP175584–KP175670

	Segura-Garcia and Yain Tun (2018)*	MH235670
	Wainwright et al. (2018)	MH243244, MH243247 and MH243249
	Ward et al. (2008)	EU398908 and EU398909

[1]Sequences of *Mobular japanica* have been included under *Mobula mobular* as the former is a junior synonym of *M. mobular* and both species are conspecifics (Last et al., 2016; White et al., 2017)

Mobula tarapacana	Bineesh et al. (2016)	KF899576–KF899580
	Chuang et al. (20016)	KP719339
	Gargan et al. (2017)	KY454873
	International Barcode of Life (2010)*	GU673481
	Li et al. (2016)*	KX060791–KX060795
	Poortvliet et al. (2015)	KM364901–KM364903
	Wainwright et al. (2018)	MH243238 and MH243252
	Ward et al. (2008)	EU398910–EU398913
Mobula thurstoni	Chuang et al. (2016)	KP719340
	da Silva Ferrette et al. (2019)	MK085566, MK085584 and MK085596
	International Barcode of Life (2010)*	GU673712, GU673713, GU674394 and GU674398
	John et al. (2020)	MG792109 and MG792121
	Li et al. (2016)*	KX060796
	Lim et al. (2015)	KM073012
	Poortvliet et al. (2015)	KM364904–KM364906
	Segura-Garcia and Yain Tun (2018)*	MH235673 and MH235674
	Wainwright et al. (2018)	MH243242, MH243243, MH243245, MH243246, MH243250, MH243251, MH243255, MH243258 and MH243259
	Ward et al. (2008)	EU398914–EU398918

	Zacharia et al. (2016)*	KX063641
Neotrygon indica	Arlyza et al. (2013a)	JX263421
	Bineesh e al. (2016)	HM467799 and KF899609–KF899613
	Borsa et al. (2013)	KC295416
	Borsa et al. (2016)	KU498035–KU498038
	Kumar et al. (2015)	JX978329
	Puckridge et al. (2013)	KC249906
	Santhanakrishnan et al. (2015)*	KT794001
Neotrygon kuhlii	Arlyza et al. (2013b)	JX304892–JX304915
	Borsa et al. (2016)	KU497940–KU497945 and KU497952–KU497960
Pateobatis jenkinsii	Ahmed et al. (2021)	MH230946
	Bineesh et al. (2016)	KF913237 and KF913238
	Cerutti-Pereyra et al. (2012)	JQ765516–JQ765518
	International Barcode of Life (2010)*	GU673708
	John et al. (2020)	MG792059, MG792066, MG792068, MG792094–MG792096
	Lim et al. (2015)	KM072991– KM072993
	Ravi et al. (2019)*	MK422144
	Segura-Garcia and Yain Tun (2018)*	MH235683
	Ward et al. (2005)	DQ108168 and DQ108169
	Ward et al. (2008)	EU398850 and EU398851
Pteroplatytrygon violacea	Bentley and Wiley (2013)*	KF930342
	Bineesh et al. (2016)	KF899654–KF899658 and HM239671
	Cariani et al. (2017)	KT307370–KT307373

	da Silva Ferrette et al. (2019)	MK085554, MK085585, MK085660, MK085682, MK085718 and MK085720
	da Silva Rodrigues Filho et al. (2020)	MN105751
	Hastings and Burton (2010)*	GU440486
	Iglesias et al. (2013)*	KF808209
	International Barcode of Life (2010)*	GU674230
	Marín et al. (2018)	MH194458
	Ramírez-Amaro et al. (2018)	KY949117–KY949119
	Song and Kim (2017)*	MG573151
Taeniura lymma	Azmir et al. (2017)	KY849556
	Bentley and Wiley (2013)*	KF930495
	Cerutti-Pereyra et al. (2012)	JQ765547–JQ765553, JQ815396 and JQ929048
	John et al. (2020)	MG792060 and MG774926
	Lim et al. (2015)	KM073026 and KM073027
	Puckridge et al. (2013)	KC250631 and KC250633
	Steinke et al. (2009)	FJ584168–FJ584170

Table S3. Diversity indices for shark species, using the mitochondrial cytochrome C oxidase subunit I: *n*, sample size; *h*, haplotype diversity; *π*, nucleotide diversity; HAP, number of haplotypes observed; UHAP, number of unique haplotypes; DHAP, number of dominant haplotypes observed; SHAP, number of shared haplotypes other than the dominant haplotype.

Species name	Topology	*h*	*π*	HAP	UHAP	DHAP	SHAP
A. pelagicus (n=146)	Complex star	0.577 ± 0.043	0.005 ± 0.0005	14	9	1 (Hap1)	4
A. superciliosus (n=104)	Star	0.164 ± 0.049	0.0005 ± 0.0001	6	5	1 (Hap1)	-
A. vulpinus (n=44)	Star	0.40 ± 0.093	0.015 ± 0.006	5	4	1 (Hap1)	-
Ca. altimus (n=29)	Simple linear	0.64 ± 0.092	0.002 ± 0.0004	7	5	1 (Hap3)	1
Ca. amblyrhynchoides (n=32)	Complex star	0.659 ± 0.083	0.004 ± 0.001	8	5	-	3
Ca. amboinensis (n=72)	Star	0.108 ± 0.049	0.002 ± 0.0011	3	2	1 (Hap1)	-
Ca. brevipinna (n=132)	Star	0.34 ± 0.054	0.005 ± 0.0013	14	11	1 (Hap1)	2
Ca. dussumieri (n=24)	Simple exclusive	0.594 ± 0.083	0.027 ± 0.0039	5	4	-	1
Ca. falciformis (n=116)	Complex mutational	0.386 ± 0.055	0.003 ± 0.001	9	7	1 (Hap5)	1
Ca. leucas (n=66)	Star	0.30 ± 0.07	0.003 ± 0.0007	4	3	1 (Hap1)	-
Ca. limbatus (n=78)	Complex mutational	0.535 ± 0.063	0.018 ± 0.0084	6	5	1 (Hap1)	-
Ca. longimanus (n=30)	Star	0.179 ± 0.088	0.0003 ± 0.0001	3	2	1 (Hap1)	-
Ca. macloti (n=12)	Star	0.561 ± 0.015	0.002 ± 0.00053	4	3	-	1
Ca. melanopterus (n=54)	Complex mutational	0.382 ± 0.078	0.003 ± 0.0012	4	1	1 (Hap1)	2
Ca. plumbeus (n=48)	Simple linear	0.671 ± 0.033	0.002 ± 0.0002	4	1	-	3
Ca. sealei (n=18)	Star	0.529 ± 0.001	0.003 ± 0.00094	4	3	-	4
Ca. sorrah (n=124)	Complex mutational	0.181 ± 0.046	0.002 ± 0.001	5	3	1 (Hap1)	1
Carcharias taurus	Complex mutational	0.535 ± 0.023	0.015± 0.0005	5	3	1 (Hap2)	1
Carcharodon carcharias (n=18)	Simple	0.004 ± 0.066	0.003 ± 0.0003	5	2	-	3
Ce. maximus (n=56)	Star	0.449 ± 0.081	0.001 ± 0.00035	5	2	-	2
Ch. griseum (n=12)	Single	0.15 ± 0.126	0.003 ± 0.0059	1	1	-	-
Ch. indicum (n=14)	Simple	0.527 ± 0.064	0.0008 ± 0.0001	2	2	-	-
Ch. punctatum (n=20)	Star	0.826 ± 0.061	0.005 ± 0.0001	8	7	1 (Hap 4)	-
G. cuvier (n=228)	Star	0.44 ± 0.024	0.002 ± 0.0002	3	1	1 (Hap2)	1
I. oxyrinchus (n=140)	Complex star	0.785 ± 0.024	0.009 ± 0.0005	17	10	-	7

I. paucus (n=46)	Star	0.63 ± 0.039	0.002 ± 0.0002	4	1	-	3
L. ditropis (n=15)	Star	0.91 ± 0.047	0.003 ± 0.0089	9	9	-	-
L. nasus (n=81)	Complex star	0.844 ± 0.034	0.007 ± 0.0089	23	19	-	4
N. acutidens (n=31)	Simple linear	0.701 ± 0.034	0.003 ± 0.0019	4	3	-	1
N. brevirostris (n=11)	Single	0	0	1	-	1 (Hap1)	-
P. glauca (n=534)	Single	0	0	1	-	1 (Hap1)	-
Rhin. typus (n=48)	Simple	0.12 ± 0.061	0.0002 ± 0.0001	2	-	1 (Hap1)	1
Rhiz. acutus (n=78)	Complex mutational	0.461 ± 0.049	0.0004 ± 0.0005	5	3	1 (Hap2)	1
Rhiz. oligolinx (n=15)	Complex star	0.02 ± 0.142	0.119 ± 0.0079	3	2	1 (Hap1)	-
S. laticaudus (n=27)	Simple	0.74 ± 0.062	0.047 ± 0.024	6	4	-	2
Sp. lewini (n=323)	Complex mutational	0.53 ± 0.026	0.023 ± 0.001	12	9	1 (Hap 3)	2
Sp. mokarran (n=59)	Complex mutational	0.1 ± 0.053	0.0007 ± 0.0004	4	3	1 (Hap1)	-
Sp. zygaena (n=91)	Single	0	0	1	-	1 (Hap1)	-
St. fasciatum (n=26)	Star	0.46 ± 0.116	0.001 ± 0.0004	5	4	1 (Hap1)	-
T. obesus (n=53)	Star	0.428 ± 0.075	0.0012 ± 0.0003	5	3	1 (Hap2)	1

Table S4. Diversity indices for ray species, based on the mitochondrial cytochrome C oxidase subunit I: *n*, sample size; *h*, haplotype diversity; *π*, nucleotide diversity; HAP, number of haplotypes observed; UHAP, number of unique haplotypes; DHAP, number of dominant haplotypes observed; OHAP, number of shared haplotypes other than the dominant haplotype.

Species name	Topology	*h*	*π*	HAP	UHAP	DHAP	SHAP
A. narinari (n=29)	Star	0.25 ± 0.12	0.001 ± 0.0008	8	7	-	1
A. ocellatus (n=18)	Complex mutational	0.76 ± 0.066	0.026 ± 0.059	5	4	-	1
B. imbricata (n=23)	Complex mutational	0.838 ± 0.053	0.036 ± 0.0096	5	5	-	-
B. walga (n = 20)	Simple exclusive	0.963 ± 0.028	0.023 ± 0.0014	14	13	1 (Hap10)	-
G. micrura (n=15)	Simple exclusive	0.848 ± 0.088	0.028 ± 0.0127	9	9	-	-
G. poecilura (n=39)	Complex mutational	0.84 ± 0.047	0.042 ± 0.0087	14	13	-	1
H. leoparda (n=78)	Simple exclusive	0.9 ± 0.019	0.030 ± 0.019	20	20	-	-
H. uarnak (n=49)	Complex mutational	0.91 ± 0.0003	0.036 ± 0.0074	12	12	-	-
Mac. gerrardi	Complex mutational	0.875 ± 0.029	0.023 ± 0.003	11	9	-	2
Mob. birostris (n=16)	Star	0.61 ± 0.130	0.002± 0.0005	5	4	1(Hap 2)	-
Mob. kuhlii (n=18)	Simple	0.853 ± 0.053	0.005 ± 0.0014	7	5	-	2
Mob. mobular (n=166)	Complex star	0.51 ± 0.034	0.003 ± 0.0007	13	11	1 (Hap1)	1
Mob. tarapacana (n=23)	Star	0.17 ± 0.102	0.002 ± 0.0051	3	2	1	-
Mob. thurstoni (n=32)	Star	0.712 ± 0.037	0.003 ± 0.0003	4	2	-	2
N. indica (n=15)	Complex mutational	0.905 ± 0.084	0.011 ± 0.0012	9	9	-	-
N. kuhlii (n=59)	Complex mutational	0.8 ± 0.045	0.015 ± 0.00103	12	12	-	-
P. jenkinsii	Complex mutational	0.823 ± 0.06	0.032 ± 0.0661	6	5	1	-
P. violacea	Complex mutational	0.671 ± 0.082	0.007 ± 0.004	5	2	1 (Hap 4)	2
T. lymma (n=20)	Complex mutational	0.82 ± 0.058	0.024 ± 0.014	5	4	1 (Hap 3)	-

References

Ahmed, MS, Datta, S, Saha, T, Hossain, Z, 2021. Molecular characterization of marine and coastal fishes of Bangladesh through DNA barcodes. *Ecology and Evolution*, *11*(2), pp.3696–3709. doi10.1002/ece3.7355.

Alghozali, FA, Wijayanti, DP, Sabdono, A, 2019. Genetic diversity of scalloped hammerhead sharks (*Sphyrna lewini)* landed in Muncar Fishing Port, Banyuwangi. *Biodiversitas Journal of Biological Diversity*, *20*(4), pp.1154–1159. doi: 10.13057/biodiv/d200430.

Almerón-Souza, F, Sperb, C, Castilho, CL, Figueiredo, PI, Gonçalves, LT, Machado, R, Oliveira, LR, Valiati, VH, Fagundes, NJ. 2018. Molecular identification of shark meat from local markets in southern Brazil based on DNA barcoding: evidence for mislabeling and trade of endangered species. *Frontiers in Genetics*, *9*, p.138. doi: https://doi.org/10.3389/fgene.2018.00138

Arlyza, IS, Shen, KN, Solihin, DD, Soedharma, D, Berrebi, P, Borsa, P. 2013. Species boundaries in the *Himantura uarnak* species complex (Myliobatiformes: Dasyatidae). *Molecular Phylogenetics and Evolution*, *66*(1), pp.429–435. https://doi.org/10.1016/j.ympev.2012.09.023

Arlyza, IS, Shen, KN, Durand, JD, Borsa, P. 2013. Mitochondrial haplotypes indicate parapatric-like phylogeographic structure in blue-spotted maskray (*Neotrygon kuhlii*) from the Coral Triangle region. *Journal of Heredity*, *104*(5), pp.725–733. doi: 10.1093/jhered/est044

Andriyono, S, Alam, MJ, Kim, HW. 2020. The Jawa and Bali Island Marine Fish Molecular Identification to Improve 12S rRNA-tRNA Valin-16S rRNA Partial Region Sequences on the GenBank Database. *Thalassas: An International Journal of Marine Sciences*, *36*(2), pp.343–356. https://doi.org/10.1007/s41208-020-00196-x

Appleyard, SA, White, WT, Vieira, S, Sabub, B. 2018. Artisanal shark fishing in Milne Bay Province, Papua New Guinea: biomass estimation from genetically identified shark and ray fins. *Scientific reports*, *8*(1), pp.1-12. doi: 10.1038/s41598-018-25101-8

Azmir, IA, Esa, Y, Amin, SMN, Md Yasin, IS, Md Yusof, FZ. 2017. Identification of larval fish in mangrove areas of Peninsular Malaysia using morphology and DNA barcoding methods. *Journal of Applied Ichthyology*, *33*(5), pp.998–1006. doi: 10.1111/jai.13425

Barbuto, M, Galimberti, A, Ferri, E, Labra, M, Malandra, R, Galli, P, Casiraghi, M. 2010. DNA barcoding reveals fraudulent substitutions in shark seafood products: the Italian case of "palombo"(*Mustelus spp.*). *Food research international*, *43*(1), pp.376–381. https://doi.org/10.1016/j.foodres.2009.10.009

Bengil, EGT, Akalin, M, Kizilkaya, IT, Bengil, F. 2019. Biology of shortfin mako shark (*Isurus oxyrinchus* Rafinesque, 1810) from the Eastern Mediterranean. *Acta Aquatica Turcica*, *15*(4), pp.425–432. doi: 10.22392/actaquatr.545997

Bénard-Capelle, J., Guillonneau, V., Nouvian, C., Fournier, N., Le Loët, K., Dettai, A., 2015. Fish mislabelling in France: substitution rates and retail types. *PeerJ*, *2*, p.714. doi: 10.7287/peerj.preprints.327v2

Berthe, C, Mourier, J, Lecchini, D, Rummer, JL, Sellos, DY, Iglésias, SP. 2016. DNA barcoding supports the presence of the cryptic ocellated eagle ray, *Aetobatus ocellatus* (Myliobatidae), in French Polynesia, South Pacific. *Cybium*, *40*(2), pp.181–184. doi: https://doi.org/10.26028/cybium/2016-402-011

Berumen, ML, Roberts, MB, Sinclair-Taylor, TH, DiBattista, JD, Saenz-Agudelo, P, Isari, S, He, Song, Khalil, MT, Hardenstine RS, Tietbohl, MD, Priest, MA, Kattan, A, Coker, D. 2019. Fishes and connectivity of Red Sea coral reefs. In C. R. Voolstra & M. L. Berumen (Eds.), *Coral Reefs of the Red Sea*, pp. 157–179. Switzerland: Springer Cham. doi: https://doi.org/10.1007/978-3-030-05802-9_8

Bhagyalekshmi, V, Kumar, AB. 2021. Bycatch of non-commercial batoids in the trawl fishery of south India: Status and conservation prerequisites. *Regional Studies in Marine Science*, *44*, p.101738. doi: 10.1016/j.rsma.2021.101738

Bineesh, KK, Gopalakrishnan, A, Akhilesh, KV, Sajeela, KA, Abdussamad, EM, Pillai, NGK, Basheer, VS, Jena, JK, Ward, RD. 2016. DNA barcoding reveals species composition of sharks and rays in the Indian commercial fishery. *Mitochondrial Dna Part A*, *28*(4), pp.458–472. doi: 10.3109/19401736.2015.1137900

Borsa, P, Arlyza, IS., Chen, WJ, Durand, JD, Meekan, MG, Shen, KN. 2013. Resurrection of New Caledonian maskray *Neotrygon trigonoides* (Myliobatoidei: Dasyatidae) from synonymy with *N. kuhlii*, based on cytochrome-oxidase I gene sequences and spotting patterns. *Comptes Rendus Biologies*, *336*(4), pp.221–232. doi: 10.1016/j.crvi.2013.02.005

Borsa, P, Shen, KN, Arlyza, IS, Hoareau, TB. 2016. Multiple cryptic species in the blue-spotted maskray (Myliobatoidei: Dasyatidae: *Neotrygon spp.*): an update. *Comptes rendus biologies*, *339*(9–10), pp.417–426. doi: 10.1016/j.crvi.2016.07.004

Cardeñosa, D., Hyde, J., Caballero, S., 2014. Genetic diversity and population structure of the pelagic thresher shark (*Alopias pelagicus*) in the Pacific Ocean: evidence for two evolutionarily significant units. *PloS one*, *9*(10), p.110193. doi: 10.1371/journal.pone.0110193

Cariani, A, Messinetti, S, Ferrari, A, Arculeo, M, Bonello, JJ, Bonnici, L, Cannas, R, Carbonara, P, Cau, A, Charilaou, C, El Ouamari, N. 2017. Improving the conservation of Mediterranean chondrichthyans: the ELASMOMED DNA barcode reference library. *PloS one*, *12*(1), p.0170244. doi: 10.1371/journal.pone.0170244

Cerutti-Pereyra, F, Meekan, MG, Wei, NWV, O'Shea, O, Bradshaw, CJ, & Austin, CM. 2012. Identification of rays through DNA barcoding: an application for ecologists. *PLoS One*, *7*(6), p.36479. doi: 10.1371/journal.pone.0036479

Chuang, PS, Hung, TC, Chang, HA, Huang, CK, Shiao, JC. 2016. The species and origin of shark fins in Taiwan's fishing ports, markets, and customs detention: A DNA barcoding analysis. *PloS one*, *11*(1), p.0147290. doi: 10.1371/journal.pone.0147290

Clark, K, Karsch-Mizrachi, I, Lipman, DJ, Ostell, J, Sayers, EW. 2016. GenBank. *Nucleic acids research*, *44*(D1), D67–D72. https://doi.org/10.1093/nar/gkv1276

da Silva Ferrette, BL, Domingues, RR, Ussami, LHF, Moraes, L, de Oliveira Magalhães, C, de Amorim, AF, Hilsdorf, AWS, Oliveira, C, Foresti, F, Mendonça, FF. 2019. DNA-based species identification of shark finning seizures in Southwest Atlantic: implications for wildlife trade surveillance and law enforcement. *Biodiversity and Conservation*, *28*(14), pp.4007–4025. doi: 10.1007/s10531-019-01862-0

da Silva Rodrigues Filho, LF, Feitosa, LM, Nunes, JLS, Palmeira, ARO, Martins, APB, Giarrizzo, T, Carvalho-Costa, LF, Monteiro, ILP, Gemaque, R, Gomes, F, Souza, RFC. 2020. Molecular identification of ray species traded along the Brazilian Amazon coast. *Fisheries Research*, *223*, p.105407. doi: 10.1016/j.fisheres.2019.105407

de Carvalho, CBV. 2014. DNA barcoding in forensic vertebrate species identification. *Brazilian Journal of Forensic Sciences, Medical Law and Bioethics*, *4*(1), pp. 12–23. http://dx.doi.org/10.17063/bjfs4(1)y201412

Deeds, JR, Handy, SM, Fry Jr. F, Granade, H, Williams, JT, Powers, M, Shipp, R, Weigt, LA. 2014. Protocol for building a reference standard sequence library for DNA-based seafood identification. *Journal of AOAC International*, *97*(6), pp.1626–1633. doi: 10.5740/jaoacint.14-111

Benson, DA, Cavanaugh, M, Clark, K, Karsch-Mizrachi, I, Lipman, DJ, Ostell, J, Sayers, EW. 2013. GenBank. Nucleic Acids Res, 41(Database issue): D36-42. doi: 10.1093/nar/gks1195. Epub 2012 Nov 27. PMID: 23193287; PMCID: PMC3531190.

Diaz-Ferguson, EE, Guzman, HM, Fahong, Y. 2016. Genetic diversity, microscale connectivity, molecular marker development and transposable elements detection in the white tip reef shark *Triaenodon obesus* (Rupell 1837). Presented at the APANAC conference, Panama City. doi: 10.13140/RG.2.2.26248.93444

Doukakis, P, Hanner, R, Shivji, M, Bartholomew, C, Chapman, D, Wong, E, Amato, G. 2011. Applying genetic techniques to study remote shark fisheries in northeastern Madagascar. *Mitochondrial DNA*, *22*(1), pp.15–20. doi: 10.3109/19401736.2010.526112

Dudgeon, CL, Broderick, D, Ovenden, JR, 2009. IUCN classification zones concord with, but underestimate, the population genetic structure of the zebra shark *Stegostoma fasciatum* in the Indo-West Pacific. *Molecular Ecology*, *18*(2), pp.248–261. doi: 10.1111/j.1365-294X.2008.04025.x

Feitosa, LM, Martins, APB, Giarrizzo, T, Macedo, W, Monteiro, IL, Gemaque, R, Nunes, JLS, Gomes, F, Schneider, H, Sampaio, I, Souza, R. 2018. DNA-based identification reveals illegal trade of threatened shark species in a global elasmobranch conservation hotspot. *Scientific reports*, *8*(1), pp.1–11. doi: 10.1038/s41598-018-21683-5

Ferrito, V, Raffa, A, Rossitto, L, Federico, C, Saccone, S, Pappalardo, AM. 2019. Swordfish or shark slice? A rapid response by COIBar–RFLP. *Foods*, *8*(11), p.537. doi: 10.3390/food8110537

Gargan, LM, Morato, T, Pham, CK, Finarelli, JA, Carlsson, JE, Carlsson, J. 2017. Development of a sensitive detection method to survey pelagic biodiversity using eDNA and quantitative PCR: A case study of devil ray at seamounts. *Marine Biology*, *164*(5), p.112. doi: 10.1007/s00227-017-3141-x

Giovos, I, Arculeo, M, Doumpas, N, Katsada, D, Maximiadi, M, Mitsou, E, Paravas, V, Aga-Spyridopoulou, RN, Stoilas, VO, Tiralongo, F, Tsamadias, IE. 2020. Assessing multiple sources of data to detect illegal fishing, trade and mislabelling of elasmobranchs in Greek markets. *Marine Policy*, *112*, p.103730. doi: 10.1016/j.marpol.2019.103730

Habib, KA, Kim, CG, Nahar, N, Akter, S, Sathi, J, Neogi, AK, Lee, YH, Oh, J, Haque, K.I. 2017a. Assessing diversity of brackish water and marine organisms of Sundarbans mangrove forest of Bangladesh through DNA barcoding. *Genome*, *60*(11), pp.940–941. doi: https://doi.org/10.1139/gen-2017-0256

Haque, AB, Das, SA, Biswas, AR. 2019. DNA analysis of elasmobranch products originating from Bangladesh reveals unregulated elasmobranch fishery and trade on species of global conservation concern. *PloS one*, *14*(9), p.0222273. doi: 10.1371/journal.pone.0222273

Hartoko, A, Pringgenies, D, Anggelina, AC, Matsuishi, T. 2020. Morphology and molecular biology of benthic Java Sea shark ray *Rhina ancylostoma* Bloch and Scheider, 1801 (Elasmobranchia: Rhinidae). *Annual Research & Review in Biology*, pp.19–31. doi: 10.9734/arrb/2020/v35i430208

Hastings, PA, Burton, RS. 2008. Establishing a DNA sequence database for the marine fish Fauna of California.

Jabado, RW, Al Ghais, SM, Hamza, W, Shivji, MS, Henderson, AC. 2015a. Shark diversity in the Arabian/Persian Gulf higher than previously thought: insights based on species composition of shark landings in the United Arab Emirates. *Marine Biodiversity*, *45*(4), pp.719–731. doi: 10.1007/s/12526-014-0275-7

Jabado, RW, Al Ghais, SM, Hamza, W, Henderson, AC, Spaet, JL, Shivji, MS, Hanner, RH. 2015b. The trade in sharks and their products in the United Arab Emirates. *Biological Conservation*, *181*, pp.190–198. doi: 10.1016/j.biocon.2014.10.032

John, AB, Asrul, MMA, Arshaad, WM, Jalal, KCA, Sheikh, HI. 2020. In S. Trivedi, H. Rehman, S. Saggu, C. Panneerselvam, S. K. Ghosh (Eds.), *DNA Barcoding and Molecular Phylogeny* (pp. 121–136). Switzerland: Springer Nature.

Johri, S, Solanki, J, Cantu, VA, Fellows, SR, Edwards, RA, Moreno, I, Vyas, A, Dinsdale, EA. 2019. 'Genome skimming' with the MinION hand-held sequencer identifies CITES-listed shark species in India's exports market. *Scientific reports*, *9*(1), pp.1–13. doi: https://doi.org/10.1038/s41598-019-40940-9

Lakra, WS, Verma, MS, Goswami, M, Lal, KK, Mohindra, V, Punia, P, Gopalakrishnan, A, Singh, KV, Ward, RD, Hebert, P. 2011. DNA barcoding Indian marine fishes. *Molecular Ecology Resources*, *11*(1), pp.60–71. doi: 10.1111/j.1755-0998.2010.02894.x

Last, PR, White, WT. 2013. Two new stingrays (Chondrichthyes: Dasyatidae) from the eastern Indonesian Archipelago. *Zootaxa*, *3722*, pp.1–21. doi: 10.11646/zootaxa.3722.1.

White, WT, Last, PR. 2016. Devil rays: Family mobulidae. In P.R. Last, W.T. White, M.R. de Carvalho, B. Séret, M.F.W. Stehmann, J.P. Naylor (Eds.), *Rays of the World*, (p.726). Ithaca, NY: CSIRO Publishing.

Lim, KC, Lim, PE, Chong, VC, Loh, KH. 2015. Molecular and morphological analyses reveal phylogenetic relationships of stingrays focusing on the family Dasyatidae (Myliobatiformes). *PLoS One*, *10*(4), p.0120518. doi: https://doi.org/10.1371/journa;.pone.0120518

Liu, SYV, Chan, CLC, Lin, O, Hu, CS, Chen, CA. 2013. DNA barcoding of shark meats identify species composition and CITES-listed species from the markets in Taiwan. *PloS one*, *8*(11), p.79373. doi: https://doi.org/10.1371/journal.pone.0079373

Marín, A, Serna, J, Robles, C, Ramírez, B, Reyes-Flores, LE, Zelada-Mázmela, E, Sotil, G, Alfaro, R. 2018. A glimpse into the genetic diversity of the Peruvian seafood sector: Unveiling species substitution, mislabeling and trade of threatened species. *PloS one*, *13*(11), p.0206596. doi: https://doi.org/10.1371/journal.pone.0206596

McCusker, MR, Denti, D, Van Guelpen, L, Kenchington, E, Bentzen, P. 2013. Barcoding Atlantic Canada's commonly encountered marine fishes. *Molecular ecology resources*, *13*(2), pp.177–188. doi: 10.1111/1755-0998.12043

Mecklenburg, CW, Møller, PR, Steinke, D. 2011. Biodiversity of arctic marine fishes: taxonomy and zoogeography. *Marine Biodiversity*, *41*(1), pp.109–140. doi: 10.1007/s12526-010-0070-z

Moftah, M, Aziz, SHA, Elramah, S, Favereaux, A. 2011. Classification of sharks in the Egyptian Mediterranean waters using morphological and DNA barcoding approaches. *PLoS One*, *6*(11), p.27001. doi: https://doi.org/10.1371/journal.pone.0027001

Moore, AB, White, WT, Ward, RD, Naylor, GJ, Peirce, R. 2011. Rediscovery and redescription of the smoothtooth blacktip shark, *Carcharhinus leiodon* (Carcharhinidae), from Kuwait, with notes on its possible conservation status. *Marine and Freshwater Research*, *62*(6), pp.528–539. doi: 10.1071/MF10159 1323-1650/11/060528

Moore, AB, Ward, RD, Peirce, R. 2012. Sharks of the Persian (Arabian) Gulf: A first annotated checklist (Chondrichthyes: Elasmobranchii). *Zootaxa*, *3167*, pp.1–16. doi: 10.11646/ZOOTAXA.3167.1.1

Momigliano, P, Jaiteh, VF. 2015. First records of the grey nurse shark *Carcharias taurus* (Lamniformes: Odontaspididae) from oceanic coral reefs in the Timor Sea. *Marine Biodiversity Records*, *8*. doi: https://doi.org/10.1017/S1755267215000354

National Center for Biotechnology Information (NCBI)[Internet]. Bethesda (MD): National Library of Medicine (US), National Center for Biotechnology Information; [1988]–[cited 2021 July 12]. Retrieved from: https://www.ncbi.nlm.nih.gov/

Nicolè, S, Negrisolo, E, Eccher, G, Mantovani, R, Patarnello, T, Erickson, DL, Kress, WJ, Barcaccia, G. 2012. DNA barcoding as a reliable method for the authentication of commercial seafood products. *Food Technology and Biotechnology*, *50*(4), pp.387–398.

Oliveira, LM, Knebelsberger, T, Landi, M, Soares, P, Raupach, MJ, Costa, FO. 2016. Assembling and auditing a comprehensive DNA barcode reference library for European marine fishes. *Journal of fish biology*, *89*(6), pp.2741–2754. doi: 10.1111/jfb.13169

Ovenden, JR, Morgan, JA, Kashiwagi, T, Broderick, D, Salini, J. 2010. Towards better management of Australia's shark fishery: genetic analyses reveal unexpected ratios of cryptic blacktip species *Carcharhinus tilstoni* and *C. limbatus*. *Marine and Freshwater Research*, *61*(2), pp.253–262. doi: 10.1071/MF09151 1323-1650/10/020253

Pavan-Kumar, A, Gireesh-Babu, P, Suresh Babu, PP, Jaiswar, AK, Prasad, KP, Chaudhari, Raje, SG, Chakrabrorty, SK, Krishna, G, Lakra, WS. 2015. DNA barcoding of elasmobranchs from Indian Coast and its reliability in delineating geographically widespread specimens. *Mitochondrial DNA*, *26*(1), pp.92–100. doi: 10.3109/19401736.2013.823174

Persis, M, Reddy, ACS, Rao, LM, Khedkar, GD, Ravinder, K, Nasruddin, K. 2009. COI (cytochrome oxidase-I) sequence based studies of Carangid fishes from Kakinada coast, India. *Molecular biology reports*, *36*(7), pp.1733–1740. doi: 10.1007/s11033-008-9375-4

Pirog, A, Jaquemet, S, Ravigné, V, Cliff, G, Clua, E, Holmes, Hussey, NE, Nevill, JEG, Temple, AJ, Berggren, P, Vigliola, L, Magalon, H. 2019. Genetic population structure and demography of an apex predator, the tiger shark *Galeocerdo cuvier*. *Ecology and evolution*, *9*(10), pp.5551–5571. doi: 10.1002/ece3.5111

Poortvliet, M, Olsen, JL, Croll, DA, Bernardi, G, Newton, K, Kollias, O'Sullivam, J, Fernando, D, Stevens, G, Magaña, FG, Seret, B. 2015. A dated molecular phylogeny of manta and devil rays (Mobulidae) based on mitogenome and nuclear sequences. *Molecular phylogenetics and evolution*, *83*, pp.72–85. doi: 10.1016/j.ympev.2014.10.012

Puckridge, M, Last, PR, White, WT, Andreakis, N. 2013. Phylogeography of the Indo-West Pacific maskrays (Dasyatidae, *Neotrygon*): A complex example of chondrichthyan radiation in the Cenozoic. *Ecology and Evolution*, *3*(2), pp.217–232. doi: 10.1002/ece3.448

Rabaoui, L, Yacoubi, L, Sanna, D, Casu, M, Scarpa, F, Lin, YJ, Shen, K-N, Todd, CR, Arculeo, M, Qurban, MA. 2019. DNA barcoding of marine fishes from Saudi Arabian waters of the Gulf. *Journal of fish biology*, *95*(5), pp.1286–1297. doi: https://doi.org/10.1111/jfb.14130

Ramírez-Amaro, S, Ordines, F, Picornell, A, Castro, JA, Ramon, C, Massutí, E, Terrasa, B. 2018. The evolutionary history of Mediterranean Batoidea (Chondrichthyes: Neoselachii). *Zoologica Scripta*, *47*(6), pp.686–698. doi: 10.1111/zsc.12315

Ribeiro, ADO, Caires, RA, Mariguela, TC, Pereira, LHG, Hanner, R, Oliveira, C. 2012. DNA barcodes identify marine fishes of São Paulo State, Brazil. *Molecular Ecology Resources*, *12*(6), pp.1012–1020. doi: 10.1111/1755-998.12007

Richards, VP, Henning, M, Witzell, W, Shivji, MS. 2009. Species delineation and evolutionary history of the globally distributed spotted eagle ray (*Aetobatus narinari*). *Journal of heredity*, *100*(3), pp.273–283. doi: 10.1093/jhered/esp005

Sales, JBL, de Oliveira, CN, dos Santos, WCR, Rotundo, MM, Ferreira, Y, Ready, J, Sampaio, I, Oliveira, C, Cruz, VP, Lara-Mendoza, RE, da Silva Rodrigues-Filho, LF. 2019. Phylogeography of eagle rays of the genus *Aetobatus*: *Aetobatus narinari* is restricted to the continental western Atlantic Ocean. *Hydrobiologia*, *836*(1), pp.169–183. doi: 10.1007/s10750-019-3949-0

Sarmiento-Camacho, S, Valdez-Moreno, M. 2018. DNA barcode identification of commercial fish sold in Mexican markets. *Genome*, *61*(6), pp.457–466. doi: 10.1139/gen-2017-0222

Sayers EW, Agarwala R, Bolton EE, Brister JR, Canese K, Clark K, Connor, R, Fiorini, N, Funk, K, Hefferon, T, Holmes, BJ, Kim, S, Kimchi, A, Kitts, P, Lathrop, S, Lu, Z, Madden, TL, Marchler-Bauer, A, Phan, L, Schneider, VA, Schoch, C, Pruitt, KD, Ostell J. 2019 Database resources of the National Center for Biotechnology Information. *Nucleic Acids Research*, 8(Database issue), pp. D23–D28. doi: 10.1093/nar/gky1069

Sembiring, A, Pertiwi, NPD, Mahardini, A, Wulandari, R, Kurniasih, EM, Kuncoro, AW, Cahyani, DNK, Anggoro, AW, Ulfa, M, Madduppa, H, Carpenter, KE, Barber, PH, Mahardika, GN. 2015. DNA barcoding reveals targeted fisheries for endangered sharks in Indonesia. *Fisheries Research*, *164*, pp.130–134. doi: 10.1016/j.fishres.2014.11.003

Spaet, JL, Berumen, ML. 2015. Fish market surveys indicate unsustainable elasmobranch fisheries in the Saudi Arabian Red Sea. *Fisheries Research*, *161*, pp.356–364. doi: 10.1016/j.fishres.2014.08.022

Steinke, D, Zemlak, TS, Hebert, PD. 2009. Barcoding Nemo: DNA-based identifications for the ornamental fish trade. *PLoS one*, *4*(7), p.6300. doi: 10.1371/journal.pone.0006300

Steinke, D, Connell, AD, Hebert, PD. 2015. Linking adults and immatures of South African marine fishes. *Genome*, *59*(11), pp.959–967. doi: 10.1139/gen-2015-0212

Sukumaran, S, Sebastian, W, Mukundan, LP, Menon, M, Akhilesh, KV, Zacharia, PU, Gopalakrishnan, A. 2020. Molecular analyses reveal a lack of genetic structuring in the scalloped hammerhead shark, *Sphyrna lewini* (Griffith & Smith, 1834) along the Indian coast. *Marine Biodiversity*, *50*(2), pp.1–6. doi: 10.1007/s12526-020-01040-4

Toha, AHA, Dailami, M, Anwar, S, Setiawan, JB, Jentewo, Y, Lapadi, I, Sutanto, S, Aryasari, R, Ambariyanto, Runtuboi, F, Madduppa, H. 2020. The genetic relationships and Indo-Pacific connectivity of whale sharks (*Rhincodon typus*) with particular reference to mitochondrial COI gene sequences from Cendrawasih Bay, Papua, Indonesia. *Biodiversitas Journal of Biological Diversity*, *21*(5), pp.2159–2171. doi: 10.13057/biodiv/d210544

Velez-Zuazo, X, Alfaro-Shigueto, J, Mangel, J, Papa, R, Agnarsson, I. 2015. What barcode sequencing reveals about the shark fishery in Peru. *Fisheries Research*, *161*, pp.34–41. doi: 10.1016/j.fishres.2014.06.005

Vella, A, Vella, N, Schembri, S. 2017. A molecular approach towards taxonomic identification of elasmobranch species from Maltese fisheries landings. *Marine genomics*, *36*, pp.17–23. doi: 10.1016/j.margen.2017.08.008

Ward, RD, Zemlak, TS, Innes, BH, Last, PR, Hebert, PD. 2005. DNA barcoding Australia's fish species. *Philosophical Transactions of the Royal Society B: Biological Sciences*, *360*(1462), pp.1847–1857. doi: 10.1098/rstb.2005.1716

Ward, RD, Holmes, BH. 2007. An analysis of nucleotide and amino acid variability in the barcode region of cytochrome c oxidase I (*cox*1) in fishes. *Molecular Ecology Notes*, *7*(6), pp.899–907. doi: 10.1111/j.1471-8286.2007.01886.x

Ward, RD, Holmes, BH, White, WT, Last, PR. 2008. DNA barcoding Australasian chondrichthyans: results and potential uses in conservation. *Marine and Freshwater Research*, *59*(1), pp.57–71. doi: 10.1071/MF07148

Wainwright, BJ, Ip, YCA, Neo, ML, Chang, JJM, Gan, CZ, Clark-Shen, N, Huang, D, Rao, M. 2018. DNA barcoding of traded shark fins, meat and mobulid gill plates in Singapore uncovers numerous threatened species. *Conservation Genetics*, *19*(6), pp.1393–1399. doi: https://doi.org/10.1007/s10592-018-1108-1

White, WT, Corrigan, S, Yang, L, Henderson, AC, Bazinet, AL, Swofford, DL, Naylor GJP. 2017. Phylogeny of the manta and devilrays (Chondrichthyes: Mobulidae), with an updated taxonomic arrangement for the family, *Zoological Journal of the Linnean Society*, *20*, pp. 1–26. https://doi.org/10.1093/zoolinnean/zlx018.

Wong, EHK, Shivji, MS, Hanner, RH. 2009. Identifying sharks with DNA barcodes: assessing the utility of a nucleotide diagnostic approach. *Molecular Ecology Resources*, *9*, 243–256. doi: 10.1111/j.1755-0998.2009.02653.x

Wynen, L, Larson, H, Thorburn, D, Peverell, S, Morgan, D, Field, I, Gibb, K. 2009. Mitochondrial DNA supports the identification of two endangered river sharks (*Glyphis glyphis* and *Glyphis garricki*) across northern Australia. *Marine and Freshwater Research*, *60*(6), pp.554–562. doi: 10.1071/MF08201

Zemlak, TS, Ward, RD, Connell, AD, Holmes, BH, Hebert, PD. 2009. DNA barcoding reveals overlooked marine fishes. *Molecular Ecology Resources*, *9*, pp.237–242. doi: 10.1111/j.1755-0998.2009.02649.x

Zhang, JB, Hanner, R. 2011. DNA barcoding is a useful tool for the identification of marine fishes from Japan. *Biochemical Systematics and Ecology*, *39*(1), pp.31–42. doi: 10.1016/j.bse.2010.12.017

Zhang, J, Hanner, R. 2012. Molecular approach to the identification of fish in the South China Sea. *PLoS One*, *7*(2), p.30621. doi: 10.1371/journal.pone.0030621

References of unpublished sequences from GenBank

Aguilar, R, Ogburn, MB, Weigt, LA, Driskell, AC, Macdonald, KS, Hines, AH. 2020. Chesapeake Bay barcode initiative (CBBI): Fishes of the greater Chesapeake Bay. https://www.ncbi.nlm.nih.gov/nuccore/MT456029.1, MT456102.1, MT456076.1, MT455809.1, MT455794.1, MT455455.1, MT436657.1, MT455293.1, MT455246.1, MT455095.1, MT455080.1, MT455925.1, MT456066.1, MT455294.1, MT455848.1, MT455995.1, MT456051.1, MT456060.1, MT455256.1, MT456108.1, MT MT455235.1, MT455562.1, MT455748.1, MT456007.1, MT455337.1, MT455567.1, MT456041.1, MT455636.1, MT455637.1

Bamaniya, D, Lakra, WS, Kumar, PA, Gireesh, BP, Krishna, G, Sharma, N, Reang, D. 2016. DNA barcoding of selected deep sea fishes from Indian coast for accurate identification & management. https://www.ncbi.nlm.nih.gov/nuccore/KJ093271.1, KJ093272.1, KJ093273.1, KJ093274.1, KJ093275.1, KJ093276.1, KJ093278.1, KJ093277.1

Basumatary, G, De, TK, Mandal, S, Thakur, S. 2017. Molecular identification of stingrays. https://www.ncbi.nlm.nih.gov/nuccore/MF495712.1, MF495713.1, MF495714.1, MF495715.1

Bentley, AC, Wiley, EO. 2013. University of Kansas Biodiversity Institute barcoding initiative. https://www.ncbi.nlm.nih.gov/nuccore/KF930342.1, KF930495.1

Bhaskar, R, Das, MK. 2018. https://www.ncbi.nlm.nih.gov/nuccore/MK331965.1, MK331966.1

Deepak, J, Diana, B, Jenson, RV, Harikrishnan, M, Madhusoodana, K. 2013. Molecular phylogeny of genus Alopias (thresher sharks). https://www.ncbi.nlm.nih.gov/nuccore/KF700944.1, KF700945.1

Deepak, J, Jenson, RV, Diana, B, Harikrishnan, M, Madhusoodanakurup, B. 2013. A comparison of tiger shark *Galeocerdo cuvier* with a novel *Galeocerdo* species and its DNA barcoding. https://www.ncbi.nlm.nih.gov/nuccore/JN657190.1

Habib, KA, Islam, MJ, Neogi, AK, Nahar, N, Rahman, SL, Mahmud, Y. 2019. Cataloging coastal fisheries of Bangladesh using DNA barcoding. https://www.ncbi.nlm.nih.gov/nuccore/MN458374.1, MN458407.1, MF614769.1

Hacohen-Domene, A, Munguia-Vega, A, Polanco-Vasquez, F, Avalos, C. 2018. First DNA barcoding study of seafood in Guatemala reveals mislabeling and the presence of threatened sharks. https://www.ncbi.nlm.nih.gov/nuccore/MK188807.1, MK188824.1

Hastings, P, Burton, R. 2010. Marine fishes of California. https://www.ncbi.nlm.nih.gov/nuccore/GU440213.1, GU440214.1, GU440259.1, GU440260.1, GU440502.1, GU440527.1, GU440486.1

Iglesias, SP, Stephan, E, Diez, G, Lebon, PY, Mayoy, S, Frotte, L, Warneiz, A, Sellos, D. 2013. Records of rare mackerel sharks (Lamniformes) from North eastern Atlantic and Mediterranean waters: *Carcharodon carcharias*, *Mitsukurina owtoni* and *Odontaspis ferox*. https://www.ncbi.nlm.nih.gov/nuccore/KM212005.1

Iglesias, SP, Mollen, FH, Tetard, A, Sellos, DY. 2013. Continuous taxonomic confusion of cryptic stingrays *Dasyatis tortonesei* and *D. Pastinaca* in European Atlantic waters needs conservation consideration. https://www.ncbi.nlm.nih.gov/nuccore/KF808209.1

International Barcode of Life. 2010. https://www.ncbi.nlm.nih.gov/nuccore/GU673375.1, GU673386.1, GU673585.1, HM387141.1, GU674232.1, GU673374.1, HQ575754.1, HQ575767.1, GU673390.1, GU674092.1, GU674234.1, GU674235.1, GU674236.1, GU674396.1, GU674397.1, GU674399.1, GU674401.1, GU673481.1, GU673712.1, GU673713.1, GU674394.1, GU674395.1GU673708.1, GU674230.1

International Barcode of Life. 2011. https://www.ncbi.nlm.nih.gov/nuccore/HQ955940.1, HQ955948.1, JN313266.1, JN312956.1, JN313301.1, JN313260.1, JN313261.1, HQ955999.1, HQ956000.1, JN313263.1, JN313264.1, HQ956137.1, JN312503.1, JN312504.1, JN312505.1, HQ956149.1, HM909793.1

Keskin, E. 2010. Phylogenetic analysis of species belong to order Lamniformes and Hexanchiformes. https://www.ncbi.nlm.nih.gov/nuccore/HQ167643.1, HQ167639.1, HQ167637.1, HQ167642.1, HQ167640.1, HQ167641.1

Khalil, AM, Gainsford, A, van Herwerden, L. 2015. DNA barcoding of Australian fresh seafood products reveals mixture of labelling successes and areas for improvement. https://www.ncbi.nlm.nih.gov/nuccore/KU366634.1, KU366614.1, KU366624.1, KU366628.1, KU366630.1

Jamaludin, NA, Mohd-Arshaad, W. 2017. DNA barcoding of sharks . https://www.ncbi.nlm.nih.gov/nuccore/MG594062.1, MG594055.1, MG594049.1

Li, H, Gao, Y, Fang, H. 2016. DNA barcoding for gill rakers of manta and devil rays (Mobulidae) from Chinese medicine markets in Guangzhou. https://www.ncbi.nlm.nih.gov/nuccore/KX060791.1, KX060792.1, KX060793.1, KX060794.1, KX060795.1, KX060796.1

Manjusha, S, Madhusoodanakurup, B. 2010. https://www.ncbi.nlm.nih.gov/nuccore/HQ589266.1, HQ589267.1, HM990646.1, HM990647.1, HM990648.1, HM990649.1, HM99051.1, HQ589285.1, HQ589286.1

Mohd-Arshaad, W, Ali, A, Pugas, AL. 2016. Identification of rays through DNA barcoding. https://www.ncbi.nlm.nih.gov/nuccore/KX219586.1

Mwangi, EW, Ndithia, HK, Njuguna, EG, Mwaura, AN. 2019. DNA barcoding to combat wildlife crime [Kenya].https://www.ncbi.nlm.nih.gov/nuccore/MK545100.1, MK545101.1

Noorul-Azliana, J, Wahidah, MA. 2017. DNA barcoding of sharks. https://www.ncbi.nlm.nih.gov/nuccore/MG644325.1, MG644347.1, MG644275.1, MG644276.1

Song, CB, Kim, MJ. 2017. https://www.ncbi.nlm.nih.gov/nuccore/MG573151.1

Prasannakumar, C, Ajmalkhan, S, Lyla, P, Murugan, S. 2008. DNA barcoding of Bay of Bengal fishes. https://www.ncbi.nlm.nih.gov/nuccore/FJ384709.1

Ravi, RK, Venu, S, Bineesh, KK, Akhilesh, KV, Sajeela, KA, Basheer, VS, Gopalakrishnan, A. 2019. https://www.ncbi.nlm.nih.gov/nuccore/MK422126.1, MK422127.1, MK422128.1, MK422129.1, MK422130.1

Santhanakrishnan, M, Mani, P, Ramakritinan, C. 2015. https://www.ncbi.nlm.nih.gov/nuccore/KT794001.1

Segura-Garcia, I, Yain Tun, T. 2018. Unscrambling Myanmar marine fishery biodiversity using DNA barcoding. https://www.ncbi.nlm.nih.gov/nuccore/MH235615.1, MH235621.1, MH235722.1, MH235723.1, MH235645.1, MH235671.1, MH235670.1, MH235673.1, MH235674.1, MH235683.1

Sijo, VP, Nishad, M, Shirke, SS. 2019. https://www.ncbi.nlm.nih.gov/nuccore/MK492925.1

Vysakh, VG, Sandhya, S, Lakshmi, M. 2019. https://www.ncbi.nlm.nih.gov/nuccore/MN017113.1

Yambot, AV, Alcantara, SG, Templonuevo, RMT. 2012. Molecular identification of high-values fish species at Cuyo, Palawan, Philippines. https://www.ncbi.nlm.nih.gov/nuccore/KC970512.1

Yokes, MB. 2016. DNA barcoding of marine fish species from Turkish Coastline. https://www.ncbi.nlm.nih.gov/nuccore/KY176421.1, KY176701.1, KY176425.1, KY176702.1, KY176584.1, KY176708.1

Zacharia, PU, Dhaneesh, KV, Shiyas, CA, Kishore, TG, Rekha Nair, J, Sobhana, KS, Sandhya, S, Pradeep, MA. 2014. Resource assessment and barcoding of elasmobranchs. https://www.ncbi.nlm.nih.gov/nuccore/ KJ475202.1, KJ475200.1

Zacharia, PU, Ambily, MN, Shiyas, CA, Kishore, TG, Ambily, L, Sobhana, KS, Rekha, JN, Najmudeen, TM, Shoba, JK, Swatipriyanka, SD, Sujitha, T, Purushottama, GB, Ranjith, L, Muktha, M, Manoj Kumar, PP, Saravanan, R, Akhilesh, KV, Santhosh, B. 2016. Resource assessment and barcoding of elasmobranchs. https://www.ncbi.nlm.nih.gov/nuccore/KX063634.1, KX063635.1, KX063636.1, KX063638.1, KX063639, KX063641.1
